# Supplementary material for: What's Happening in Your Head: Overcoming Our Assumptions to Work Better Together
Source: MedEdPORTAL. 2020 Nov 30;16:11034. doi: 10.15766/mep_2374-8265.11034 (PMC7703482; doi:10.15766/mep_2374-8265.11034)
Supplement: Supplementary file 1 — Ladder of Inference Poster.pptxLadder of Inference Poster.docxCharacter Cards.docxSituation Cards.docxRung Concept Cards.docxLadder of Inference Presentation.pptxExercise 1 Instructions and Talking Points.docxExercise 2 Instructions and Talking Points.docxLadder of Inference Workshop Assessment Tool.docx [file mep_2374-8265.11034-s001.zip › A. Ladder of Inference Poster.pptx]

## Slide 1
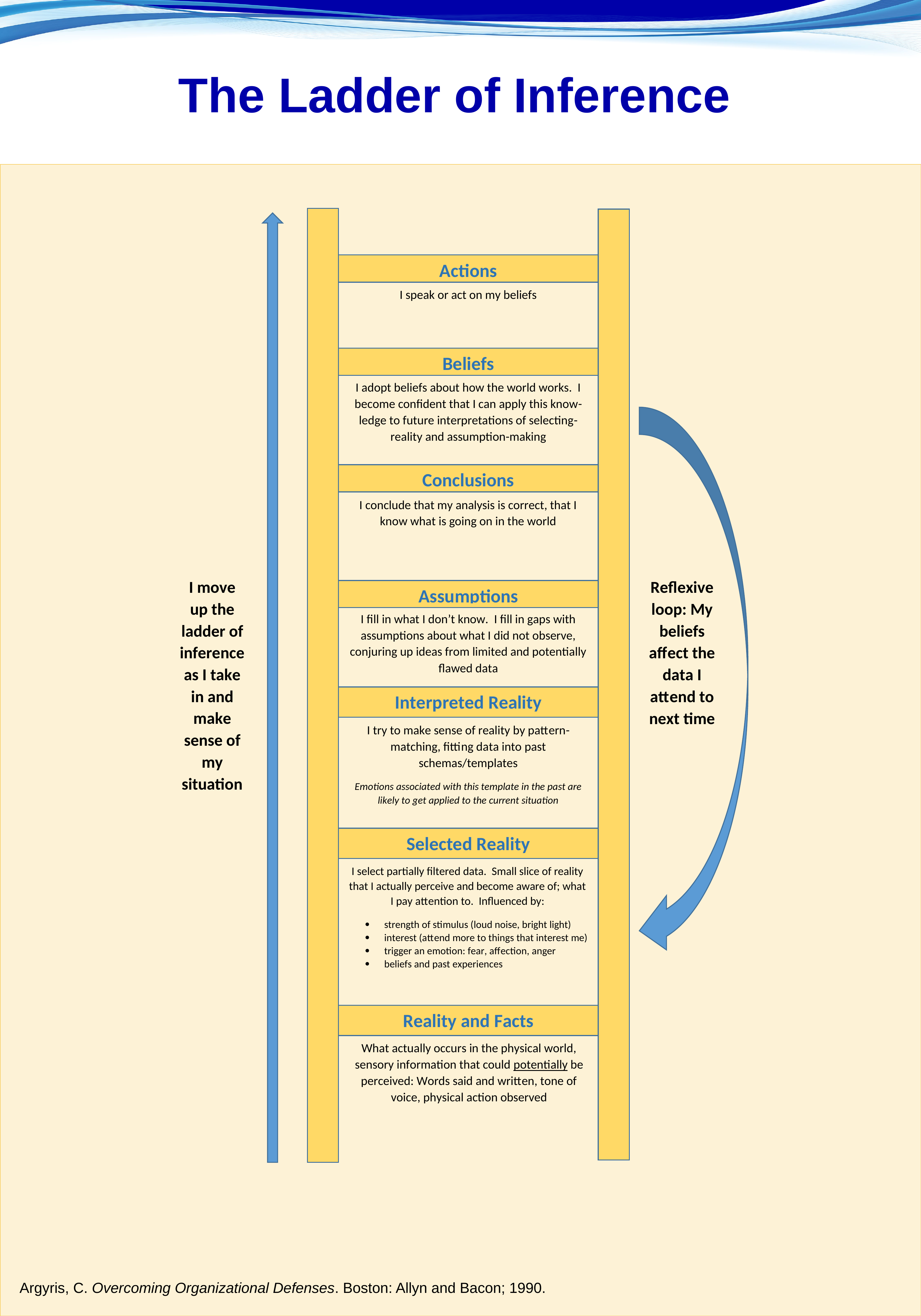

The Ladder of Inference
Argyris, C. Overcoming Organizational Defenses. Boston: Allyn and Bacon; 1990.
