# Supplementary material for: What's Happening in Your Head: Overcoming Our Assumptions to Work Better Together
Source: MedEdPORTAL. 2020 Nov 30;16:11034. doi: 10.15766/mep_2374-8265.11034 (PMC7703482; doi:10.15766/mep_2374-8265.11034)
Supplement: Supplementary file 1 — Ladder of Inference Poster.pptxLadder of Inference Poster.docxCharacter Cards.docxSituation Cards.docxRung Concept Cards.docxLadder of Inference Presentation.pptxExercise 1 Instructions and Talking Points.docxExercise 2 Instructions and Talking Points.docxLadder of Inference Workshop Assessment Tool.docx [file mep_2374-8265.11034-s001.zip › C. Character Cards.docx]

**Appendix C. Character Cards**

Each of the following pages should be printed prior to the exercise and should be held by the facilitator for distribution in Exercise 1. It is acceptable for each to be printed on 8 ½ x 11 inch plain paper, but cardstock is preferred. Printing on colored paper can add appeal but is not necessary. One Character Card will be printed and provided to each group as detailed in the other workshop instructions (at the outset of each round of Exercise 1).

Eight sample Character Cards are included here. The facilitator may modify these as desired or create as many additional cards as desired if there are more than eight groups in the workshop. The key element of any cards created *de novo* is that they contain multiple descriptors of traits or experiences of a fictitious person, and that some be things about which they are proud and others about which they are worried/anxious. The Character Cards may be modified and tailored to reflect characters or character experiences that could plausibly exist among the identity of the group’s participants. For example, these Character Cards are suited to Family Medicine Residents but could be modified to “ring true” for surgery or psychiatry residents.

**Character Background A**

PGY2 Resident

- Has requested to do a two-week rotation in Peru, something which is unusual for residents.
- Has racked up 10 on-campus parking tickets in 6 months and Security has emailed them about this.
- Is completely up to date on e-prescription renewals but has 12 unanswered e-messages, some of which are 5 days old.
- At last meeting with advisor was told that faculty were concerned about their medical knowledge.

**Character Background B**

PGY2 Resident

- Got an email from Outpatient Clinic Office Manager yesterday that a patient they cared for was very upset that a prescription was sent to the wrong pharmacy.
- Got CC’d on an email last week from Emergency Department Medical Director to their own Program Director, praising them for resuscitating a very ill patient.
- Did not get around to taking Step 3 as required to become a PGY2 resident…but no one seems to have noticed.
- Got a notice that the poster they were working on was accepted.

**Character Background C**

PGY3 Resident

- Got a notice from Patient Relations that they will be recognized for excellent patient satisfaction scores.
- Stole two chocolate chip cookies from hospital cafeteria yesterday but is pretty sure no one noticed.
- Emailed Program Director yesterday asking them to write a letter of recommendation for fellowship.
- During rounds yesterday, dropped program-provided laptop, which now has a small crack in the screen.
- Uncle happens to be the new president/CEO of the hospital and it is known that the residency program is under budgetary review

**Character Background D**

PGY3 Resident

- Graduates in 4 months and does not have a job lined up, but met with Program Director three days ago to discuss several possibilities for employment
- Has 20 unsigned charts, 10 of which are two weeks old
- Grandmother is very ill. Cell phone doesn’t get reception in the hospital and family knows resident can be reached by calling Program Coordinator.
- While on call last night, was streaming something on work laptop that was…questionable

**Character Background E**

PGY1 Resident

- Seems to be doing great in all rotations according to faculty feedback.
- Had a really nice conversation earlier this week with the cute new medical student on service…and perhaps someone could have misunderstood it as flirting.
- Submitted a request yesterday for the Program to reimburse their recent trip to “Renew Health Spa” with the idea that experiencing massage counts as “Continuing Medical Education.”
- Brought in a bouquet of flowers to show appreciation to the program’s coordinator and administrative staff

**Character Background F**

PGY3 Resident

- While precepting with Program Director last week, had a really nice chat about their shared favorite TV show
- Got borderline-failing evaluations on inpatient rotation last year and knows things didn’t go well there last month either
- Got into an argument with a respiratory therapist who administered albuterol nebulizer every 6 hours instead of every 4 hours as the resident ordered.
- Asked a faculty member to be allowed to help plan the residency retreat this year

**Character Background G**

PGY2 Resident

- Recently announced that they would like to be Chief Resident for next year.
- In the call room last week was holding hands with the PGY1 resident they are secretly dating when a faculty member walked in and saw them.
- Was talking with other residents yesterday and didn’t realize Program Director was in the nearby hallway while saying about them “That old fool needs to retire.”
- Gave an interview about vaccinations for the local newspaper last week

**Character Background H**

PGY1 Resident

- Is doing a great job on Quality Improvement Project for residency and seems to be leading even senior residents on the team.
- Recently posted on Faceplant social media site a photo of a patient’s really nasty foot infection.
- Submitted a request for two days off later this year that would exceed the maximum allowed.
- Recently told Program Director that their best friend from medical school is a very impressive 4^th^ year student who might apply to this program.
