# Supplementary material for: What's Happening in Your Head: Overcoming Our Assumptions to Work Better Together
Source: MedEdPORTAL. 2020 Nov 30;16:11034. doi: 10.15766/mep_2374-8265.11034 (PMC7703482; doi:10.15766/mep_2374-8265.11034)
Supplement: Supplementary file 1 — Ladder of Inference Poster.pptxLadder of Inference Poster.docxCharacter Cards.docxSituation Cards.docxRung Concept Cards.docxLadder of Inference Presentation.pptxExercise 1 Instructions and Talking Points.docxExercise 2 Instructions and Talking Points.docxLadder of Inference Workshop Assessment Tool.docx [file mep_2374-8265.11034-s001.zip › D. Situation Cards.docx]

**Appendix D. Situation Cards**

Each of the following pages should be printed prior to the exercise and should be held by the facilitator for distribution in Exercise 1. It is acceptable for each to be printed on 8 ½ x 11 inch plain paper, but cardstock is preferred. Printing on colored paper can add appeal but is not necessary. One Situation Card will be printed and provided to each group as detailed in the other workshop instructions (at the outset of each round of Exercise 2).

Eight sample Situation Cards are included here. The facilitator may modify these as desired or create as many additional cards as desired if there are more than eight groups in the workshop. The key element of any cards created *de novo* is that they describe an ambiguous, preferably emotionally-charged situation that could be interpreted multiple ways. For example, these Situation Cards are suited to Family Medicine Residents (or to people in general) but could be modified to “ring true” for surgery or psychiatry residents.

**Situation A**

**Reality and Facts + Selected Reality**

You are driving to work, keeping up with traffic in the left lane of the highway. Suddenly a car passes you on the right and pulls in the small space between you and the car ahead of you. You step on the brakes, you feel your face flush and your pulse pound as at the same moment you notice a bumper sticker on the car, advertising the name of the person you most certainly did NOT vote for in the last presidential election.

**Situation B**

**Reality and Facts + Selected Reality**

It’s been nice living in the same town for high school, college, and medical school, with family and friends you are very close with. You open you Match Day envelope and learn that you are leaving them for a residency on the other side of the country. You text the big news to your best friend, who replies:

“can’t talk – l8r”

(there is no emoji)

**Situation C**

**Reality and Facts + Selected Reality**

You are a residency faculty member. Following your weekly faculty meeting, your Program Director pulls you into their office and hands something to you, saying that they found it on their desk this morning. It is an unsigned note addressed to the Program Director which reads “Because of their unprofessional behavior and poor medical knowledge, Dr. (your name) shouldn’t be permitted to teach any more. Other residents feel this way too.”

**Situation D**

**Reality and Facts + Selected Reality**

You are the Night Float Resident, signing out to the Day Team, on which the PGY3 and PGY1 resident are known to be good friends. It was a rough night, you are exhausted emotionally and physically and there are a lot of loose ends left over – lots of work you just couldn’t get done. As you finish speaking and look up from your notes, you catch the PGY1 and PGY3 giving each other a “look.”

**Situation E**

**Reality and Facts + Selected Reality**

You are a resident and think you’re progressing on track. Your review meeting with your advisor seemed to go well 6 months ago and your preceptors often say “good job” at the end of your precepting sessions. Other than that you don’t recall particularly good or bad feedback being given to you. At your current advising meeting, your advisor shares written feedback with you that says that all the faculty reviewed your evaluations and feels you’re not doing well and you need an educational intervention plan.

**Situation F**

**Reality and Facts + Selected Reality**

That 4^th^ floor nurse you dated last year was very fun but also a bit vindictive. When you were dating, you remember them posting photos and some embarrassing comments about their “ex-.” At the time, you were glad that wasn’t you. But now you broke up, you are the new “ex-“, and there are some hard feelings.

Over the past few weeks, you notice that whenever you walk onto the 4^th^ floor, the younger nurses seem to smirk and the unit clerk Old Miss Mabel seems to frown slightly and shake her head. Nothing seems different about the other hospital floors.

**Situation G**

**Reality and Facts + Selected Reality**

In clinic today you got assigned “Sloth Shelly” as your nurse again. Your first patient got roomed late so you have been running behind all afternoon. The patients make their irritation clear to you. And your heartless preceptor “Ruthless Ruth” told you that clinic needs to be done at 5:00 promptly so they can take their child to soccer practice. You wrap up a visit and walk out of the room 30 minutes behind schedule, to see that your two exam rooms are empty, three patients are waiting to be roomed, and your nurse appears to be talking to another nurse down the hallway.

**Situation H**

**Reality and Facts + Selected Reality**

In the 2 ½ years you have known this patient, you have some degree of a rapport and trust with them. You know how chaotic their life is and that their job that barely keeps their family afloat is possible only when they are taking their opioids for their chronic pain. Nothing else has worked for their pain. They called your clinic today -- they need a refill a week early. They say their pills were in the bottle yesterday but are gone today. Maybe their son took them? They are out of sick days and if they don’t take their medicine they can’t work. If they can’t work, the financial impact would be devastating.
