# Supplementary material for: What's Happening in Your Head: Overcoming Our Assumptions to Work Better Together
Source: MedEdPORTAL. 2020 Nov 30;16:11034. doi: 10.15766/mep_2374-8265.11034 (PMC7703482; doi:10.15766/mep_2374-8265.11034)
Supplement: Supplementary file 1 — Ladder of Inference Poster.pptxLadder of Inference Poster.docxCharacter Cards.docxSituation Cards.docxRung Concept Cards.docxLadder of Inference Presentation.pptxExercise 1 Instructions and Talking Points.docxExercise 2 Instructions and Talking Points.docxLadder of Inference Workshop Assessment Tool.docx [file mep_2374-8265.11034-s001.zip › E. Rung Concept Cards.docx]

**Appendix E. Rung Concept Cards**

The pages that follow are to be printed in advance of the activity. These should be printed on 8 ½ x 11 inch paper. While plain office paper is acceptable, cardstock is preferred. The use of various colors may add interest. After printing, each page should be cut with a paper cutter such that a concept such as “Selected Reality” or “Interpreted Reality” appears as a heading on each half page, with an open field below the heading where participants will write. A set of Rung Cards is thus comprised of 5 half-pages, each with a different concept. If colored paper is used, each set should be the same color. It is recommended that an adhesive such as a two-sided adhesive strips be applied to the back of each card in advance of the activity although masking tape can be substituted.
One set of cards should be placed at each station for every round the activity will be performed, for a minimum of two sets per station (for one round of Exercise 1 and one round of Exercise 2).

**Interpreted Reality**

I try to make sense of reality by pattern-matching, fitting data into past schemas/templates

*Emotions associated with this template in the past are likely to get applied to the current situation*

**Assumptions**

I fill in what I don’t know. I fill in gaps with assumptions about what I did not observe, conjuring up ideas from limited and potentially flawed data

**Conclusions**

I conclude that my analysis is correct, that I know what is going on in the world

**Beliefs**

I adopt beliefs about how the world works. I become confident that I can apply this knowledge to future interpretations of selecting-reality and assumption-making

**Actions**

I speak or act on my beliefs
