# Supplementary material for: What's Happening in Your Head: Overcoming Our Assumptions to Work Better Together
Source: MedEdPORTAL. 2020 Nov 30;16:11034. doi: 10.15766/mep_2374-8265.11034 (PMC7703482; doi:10.15766/mep_2374-8265.11034)
Supplement: Supplementary file 1 — Ladder of Inference Poster.pptxLadder of Inference Poster.docxCharacter Cards.docxSituation Cards.docxRung Concept Cards.docxLadder of Inference Presentation.pptxExercise 1 Instructions and Talking Points.docxExercise 2 Instructions and Talking Points.docxLadder of Inference Workshop Assessment Tool.docx [file mep_2374-8265.11034-s001.zip › F. Ladder of Inference Presentation.pptx]

## Slide 1
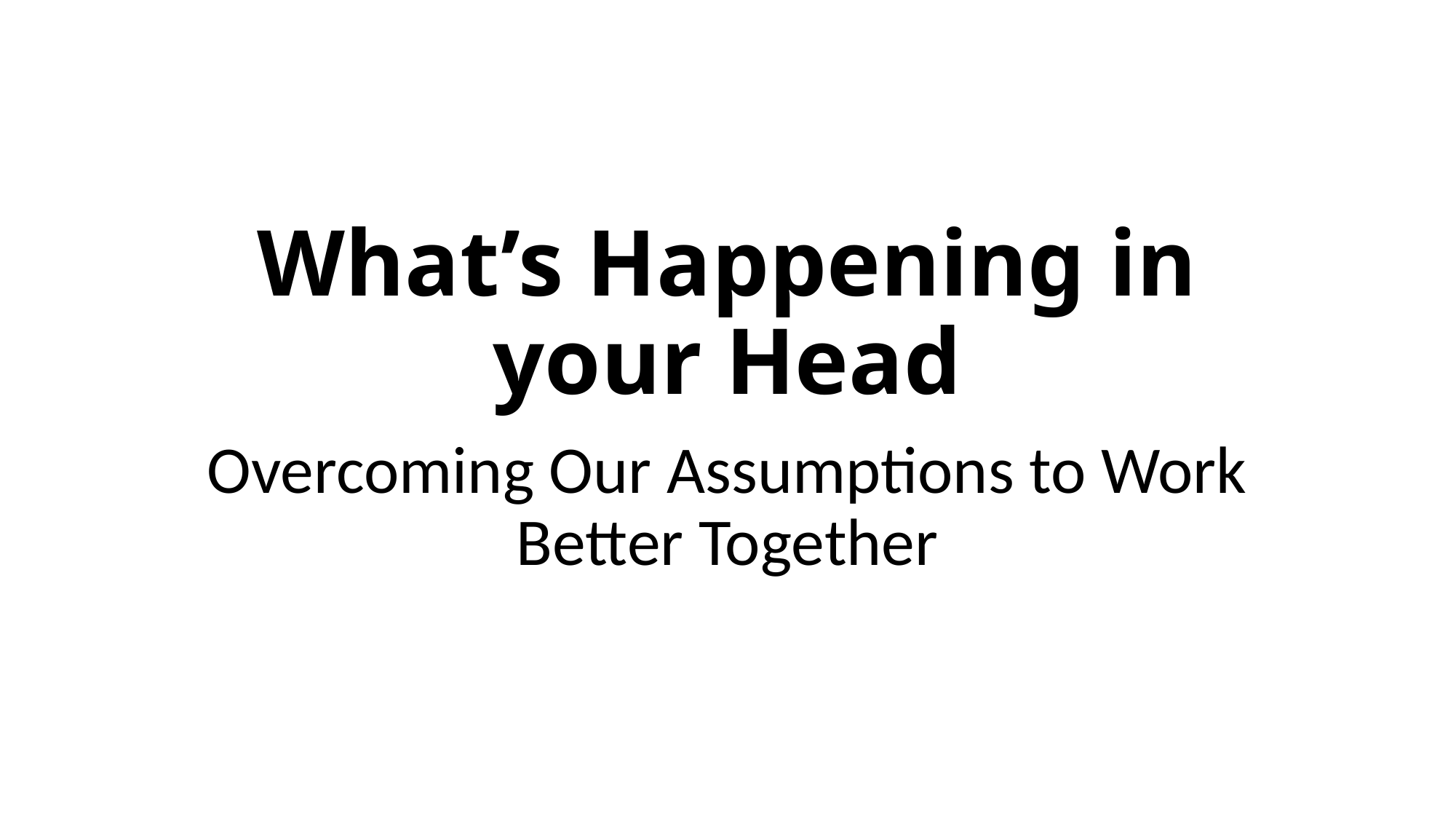

# What’s Happening in your Head
Overcoming Our Assumptions to Work Better Together

## Slide 2
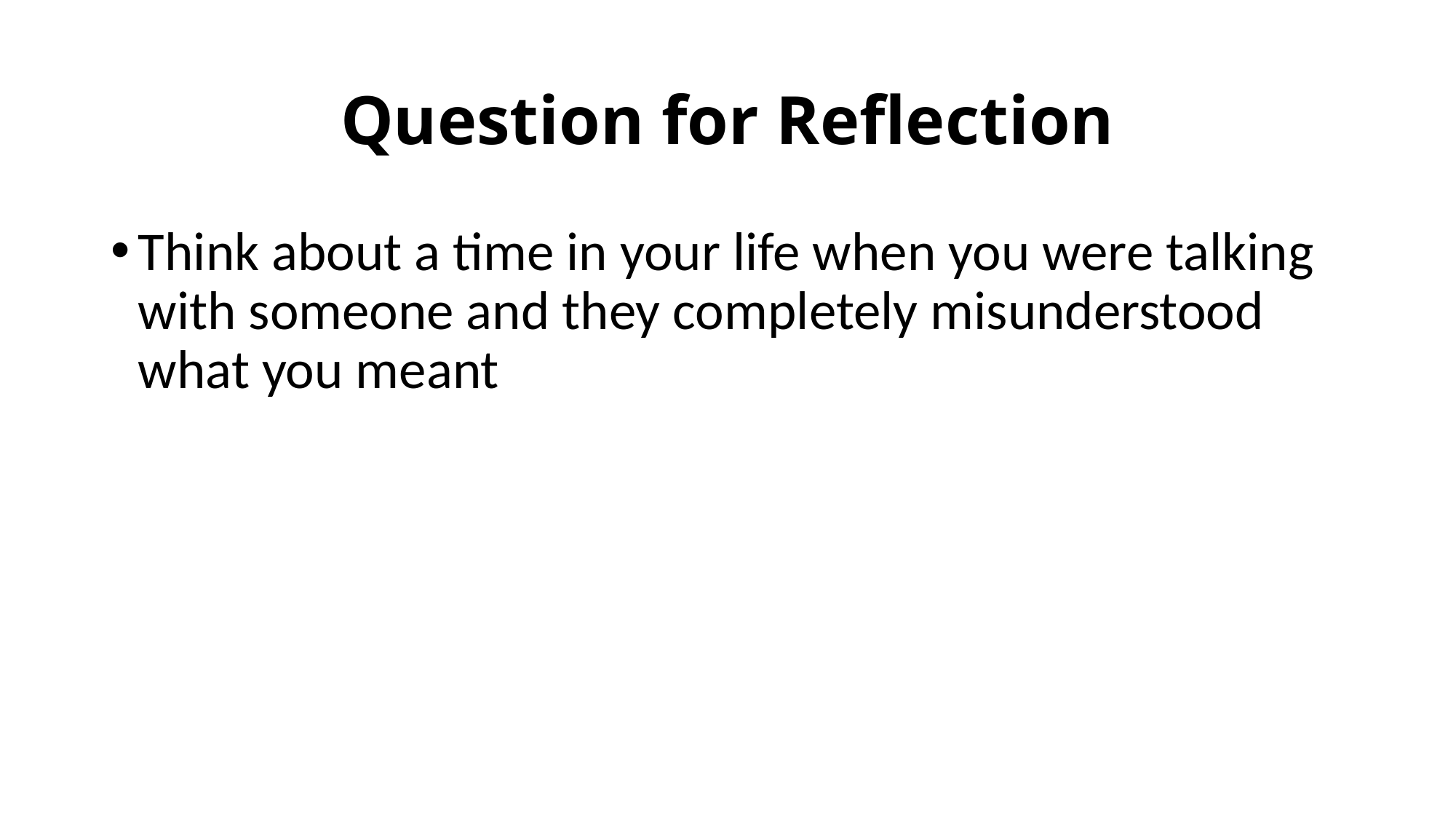

# Question for Reflection
Think about a time in your life when you were talking with someone and they completely misunderstood what you meant

## Slide 3
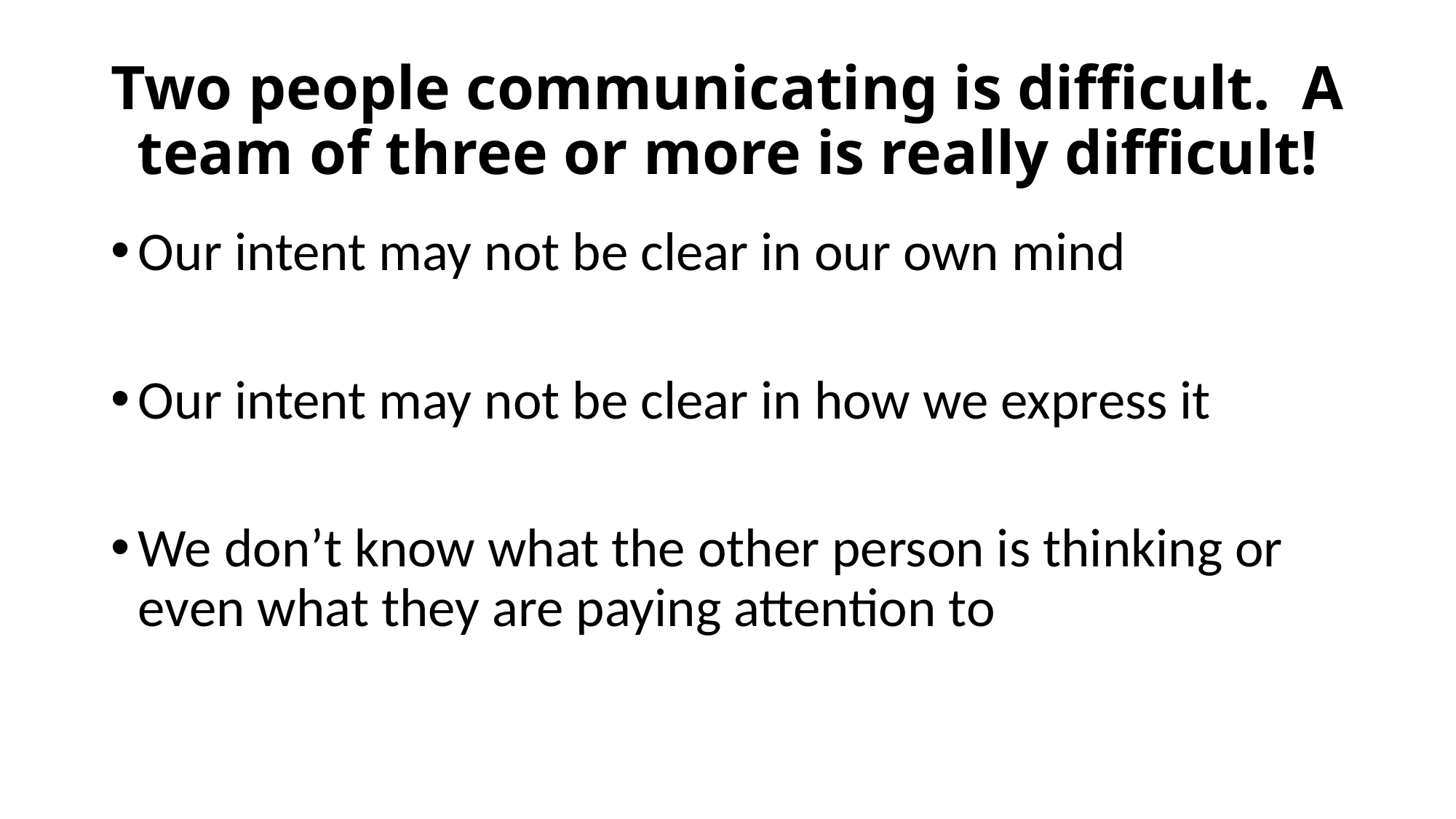

# Two people communicating is difficult. A team of three or more is really difficult!
Our intent may not be clear in our own mind
Our intent may not be clear in how we express it
We don’t know what the other person is thinking or even what they are paying attention to

## Slide 4
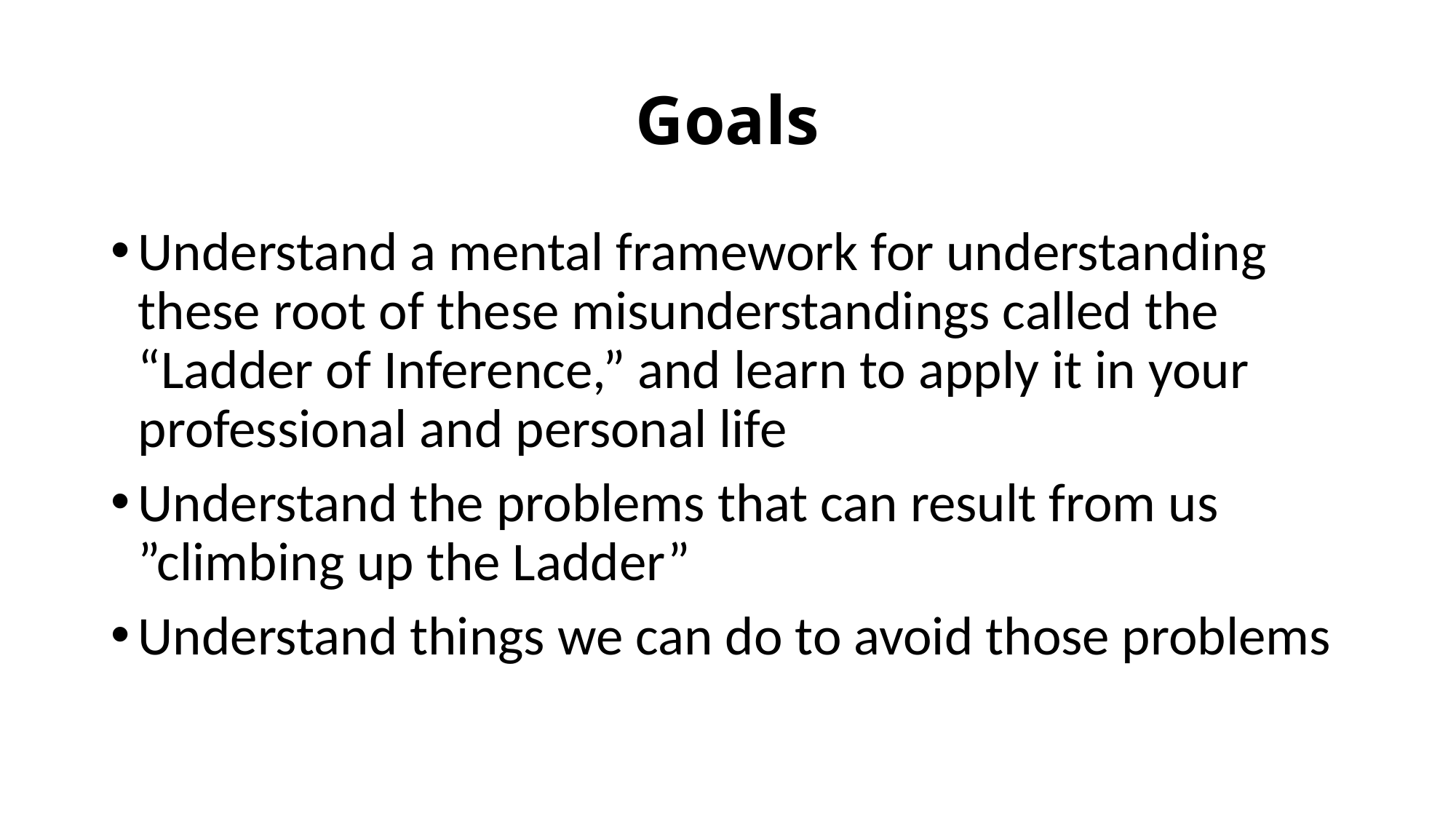

# Goals
Understand a mental framework for understanding these root of these misunderstandings called the “Ladder of Inference,” and learn to apply it in your professional and personal life
Understand the problems that can result from us ”climbing up the Ladder”
Understand things we can do to avoid those problems

## Slide 5
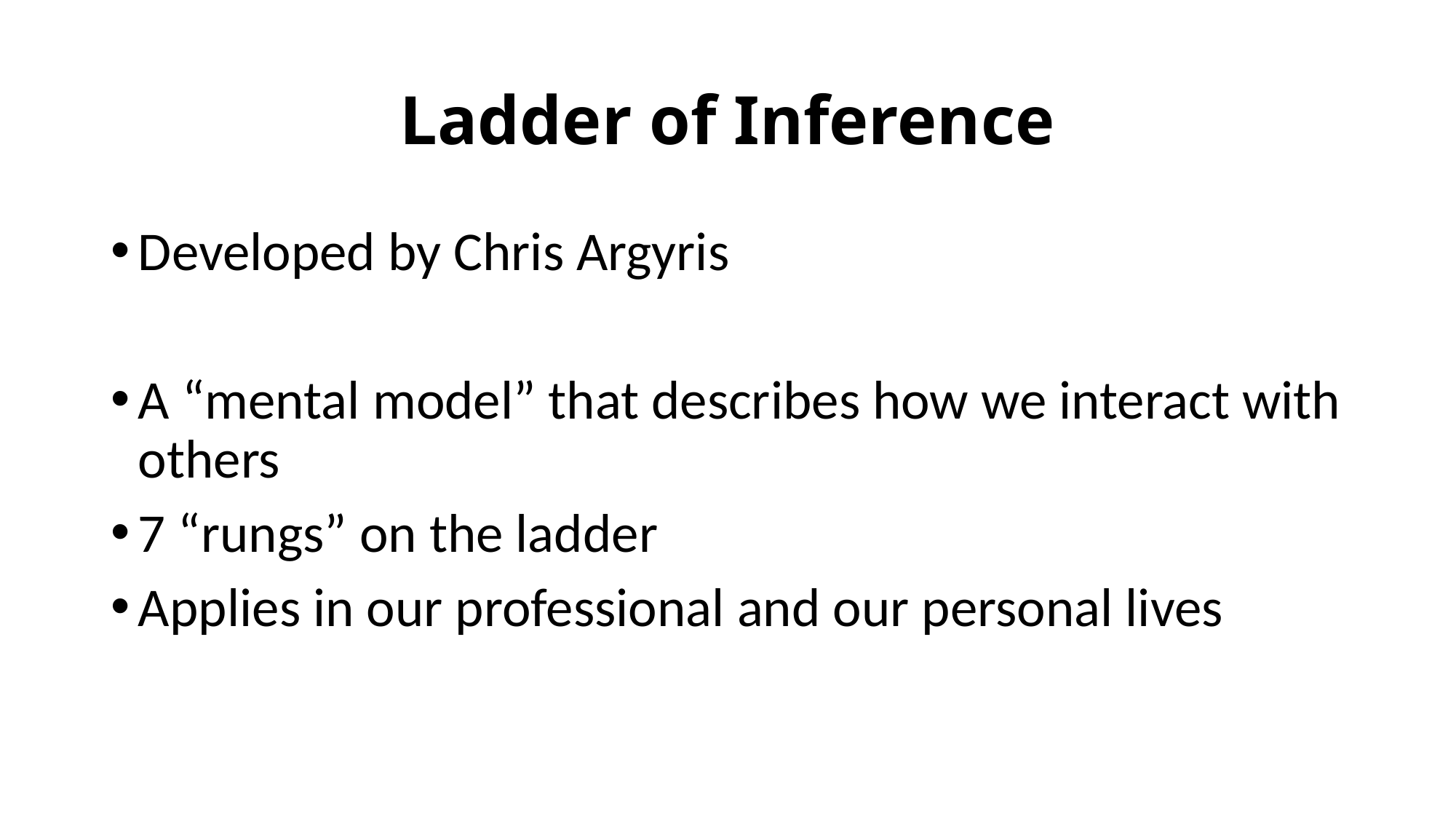

# Ladder of Inference
Developed by Chris Argyris
A “mental model” that describes how we interact with others
7 “rungs” on the ladder
Applies in our professional and our personal lives

## Slide 6
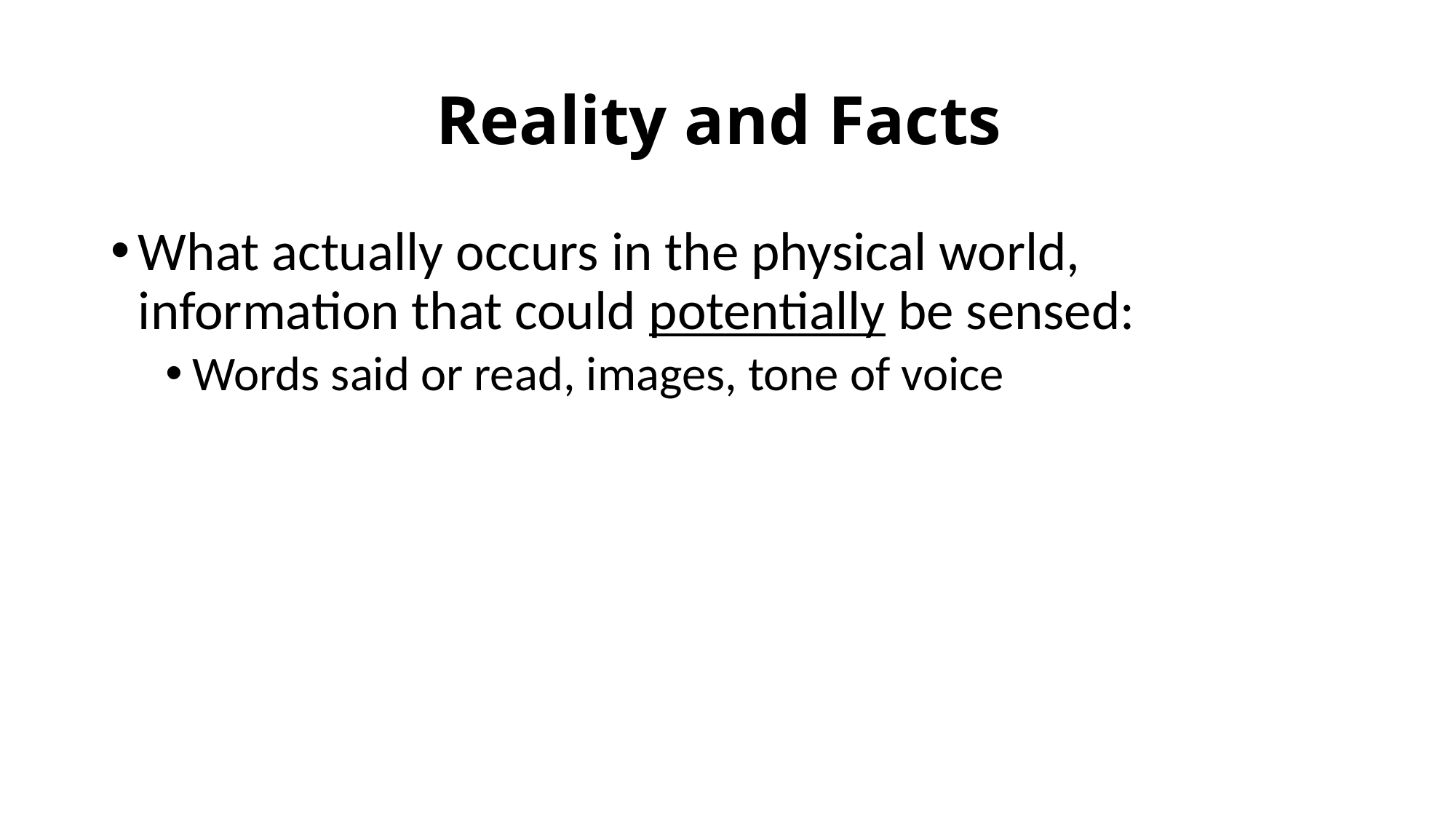

# Reality and Facts
What actually occurs in the physical world, information that could potentially be sensed:
Words said or read, images, tone of voice

## Slide 7
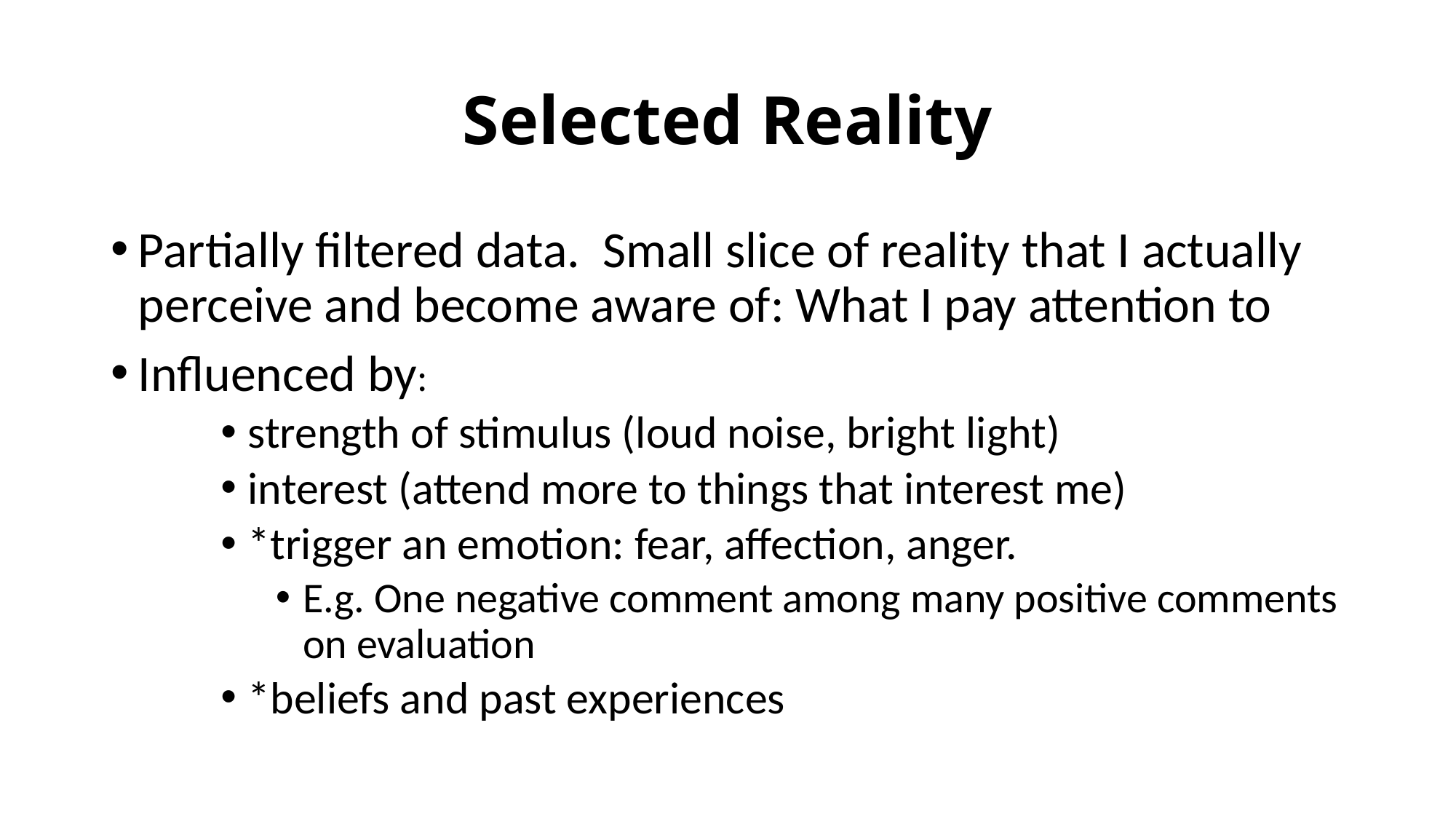

# Selected Reality
Partially filtered data. Small slice of reality that I actually perceive and become aware of: What I pay attention to
Influenced by:
strength of stimulus (loud noise, bright light)
interest (attend more to things that interest me)
*trigger an emotion: fear, affection, anger.
E.g. One negative comment among many positive comments on evaluation
*beliefs and past experiences

## Slide 8
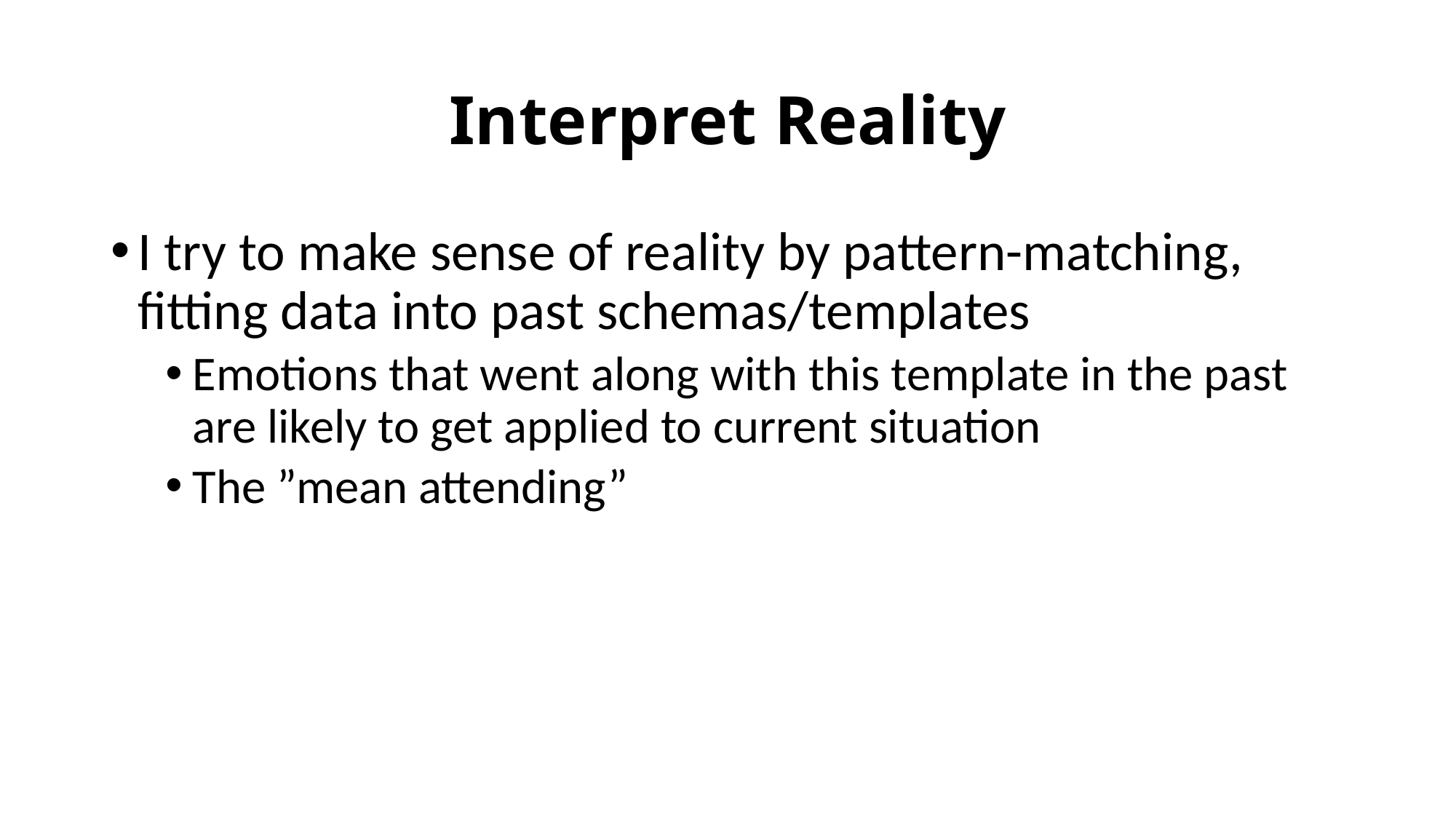

# Interpret Reality
I try to make sense of reality by pattern-matching, fitting data into past schemas/templates
Emotions that went along with this template in the past are likely to get applied to current situation
The ”mean attending”

## Slide 9
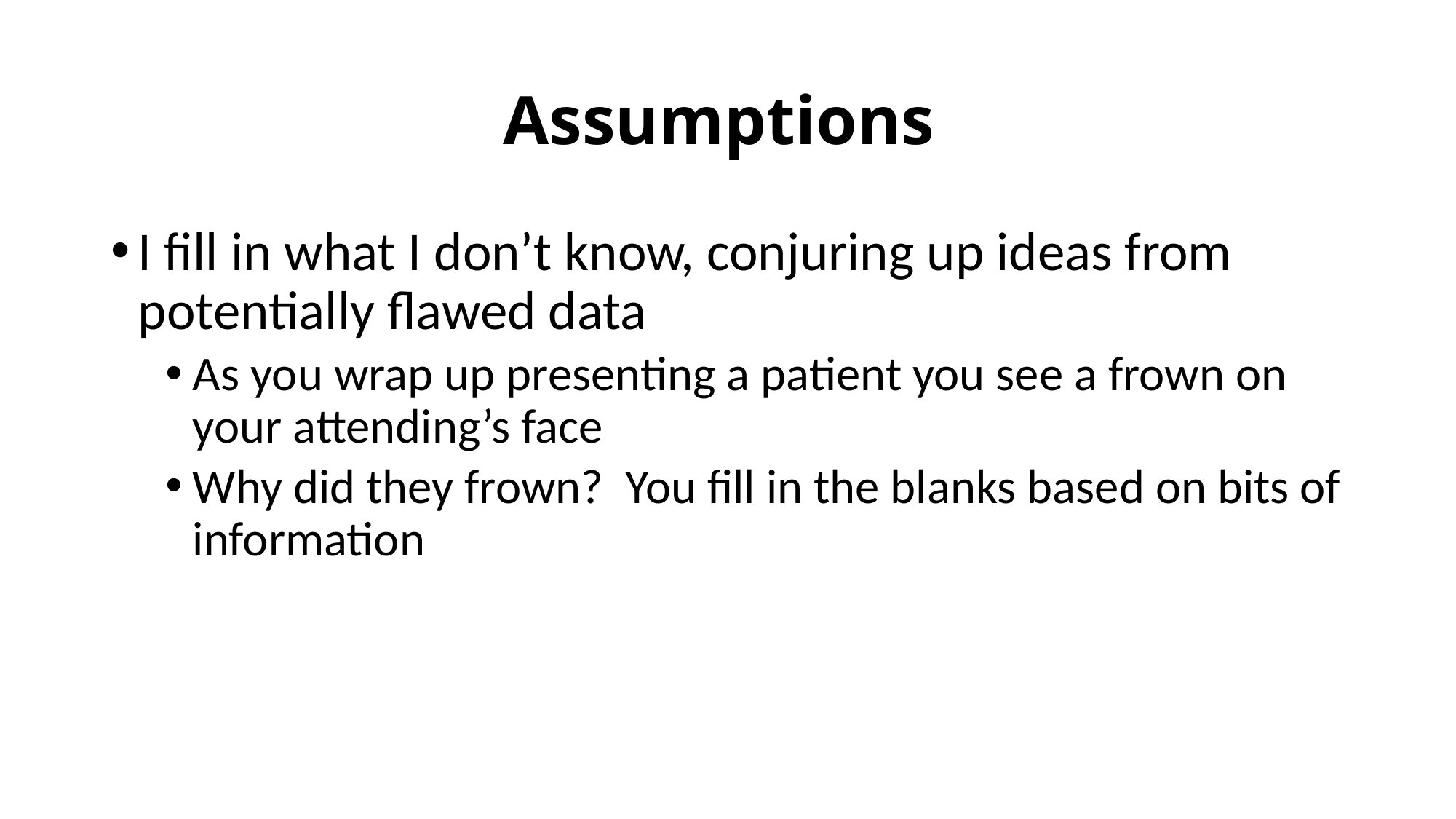

# Assumptions
I fill in what I don’t know, conjuring up ideas from potentially flawed data
As you wrap up presenting a patient you see a frown on your attending’s face
Why did they frown? You fill in the blanks based on bits of information

## Slide 10
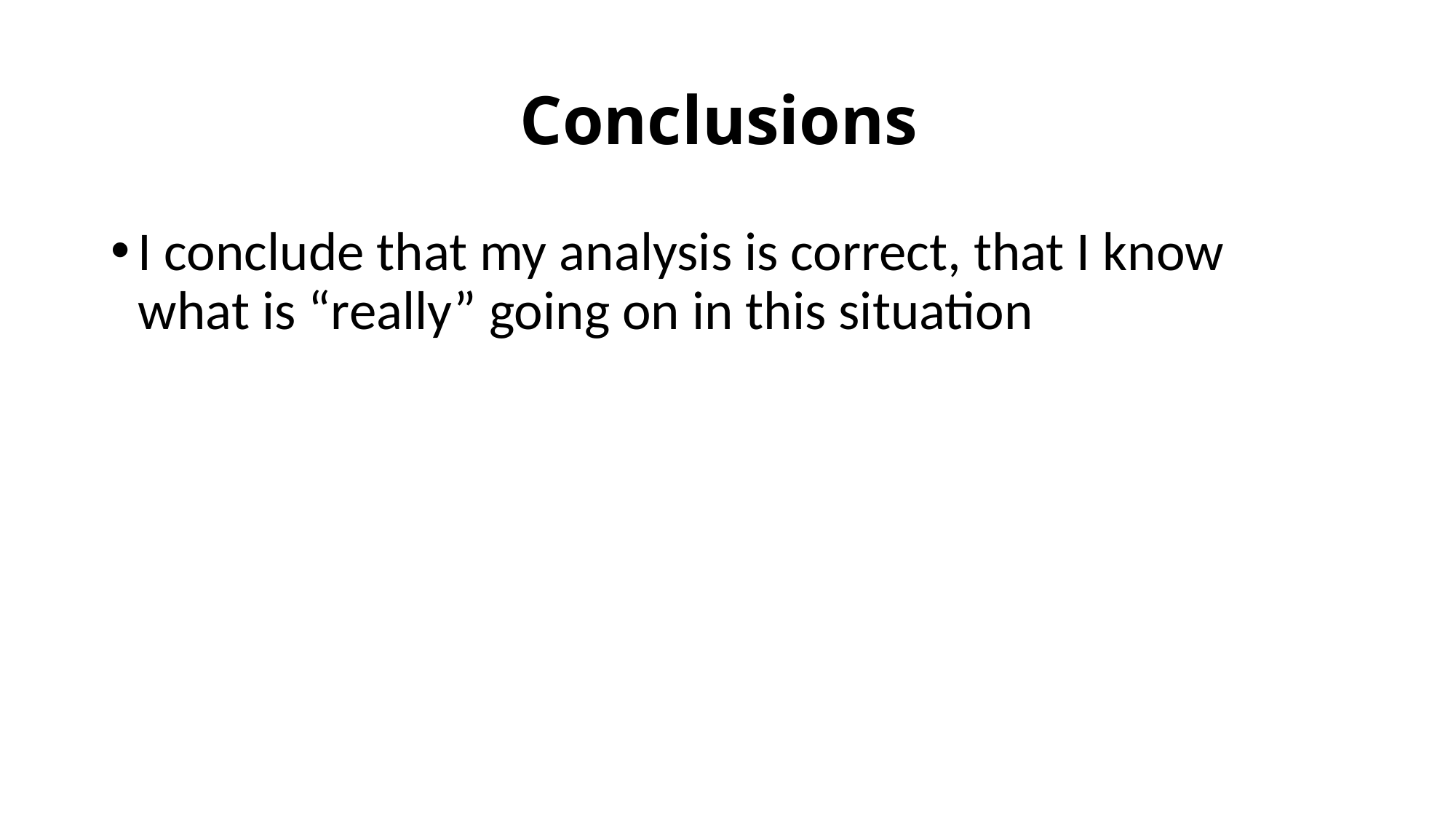

# Conclusions
I conclude that my analysis is correct, that I know what is “really” going on in this situation

## Slide 11
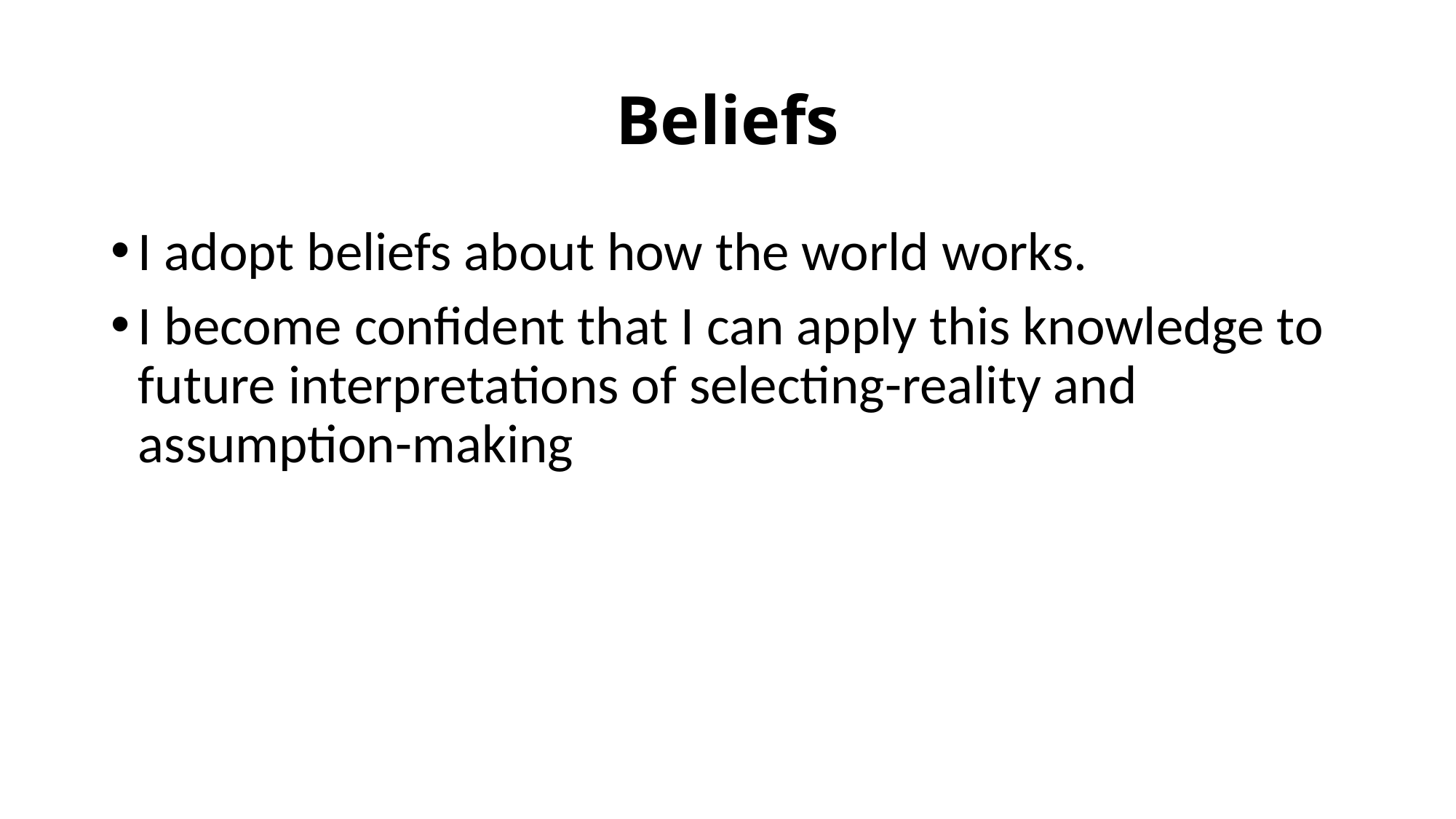

# Beliefs
I adopt beliefs about how the world works.
I become confident that I can apply this knowledge to future interpretations of selecting-reality and assumption-making

## Slide 12
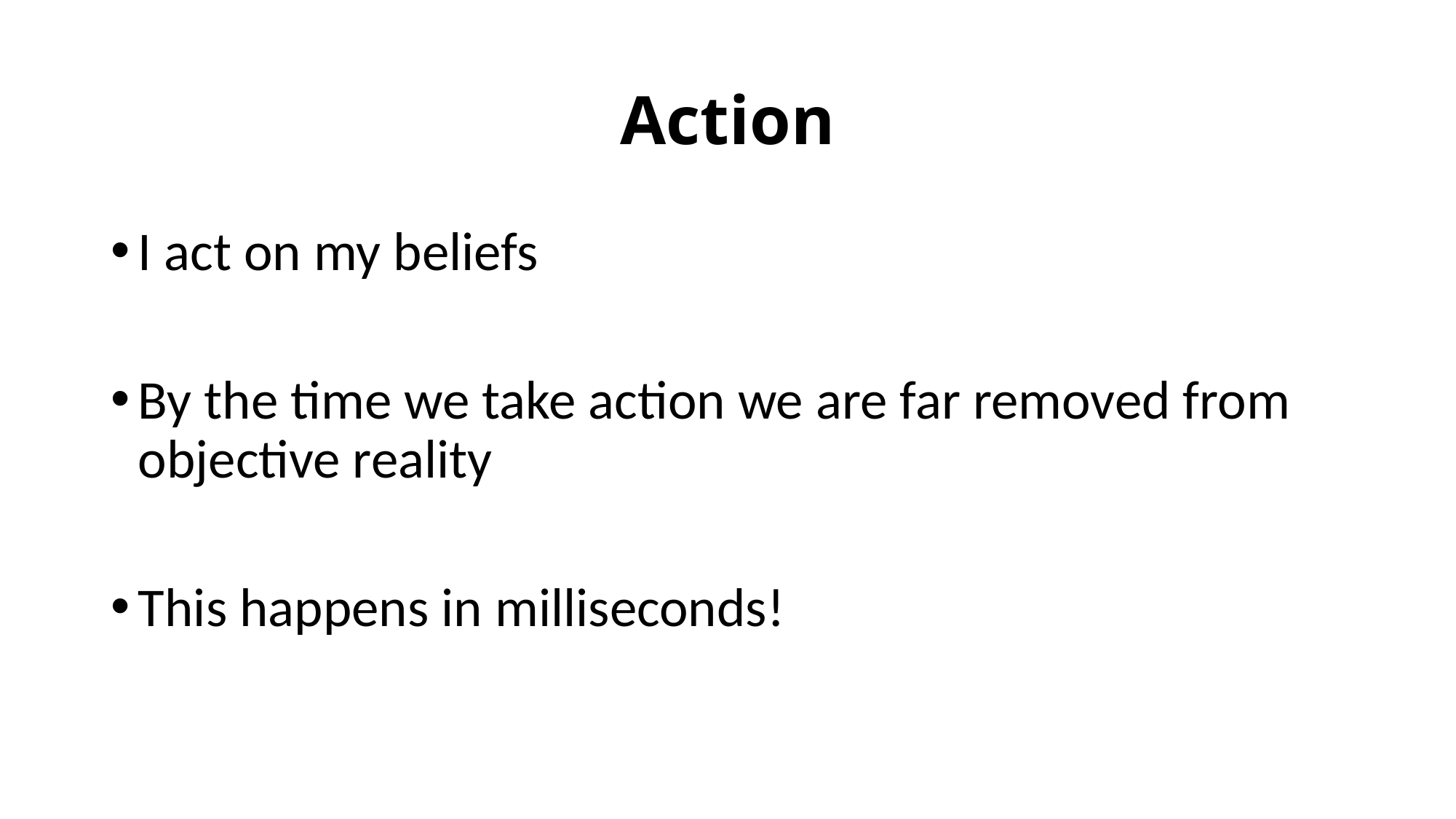

# Action
I act on my beliefs
By the time we take action we are far removed from objective reality
This happens in milliseconds!

## Slide 13
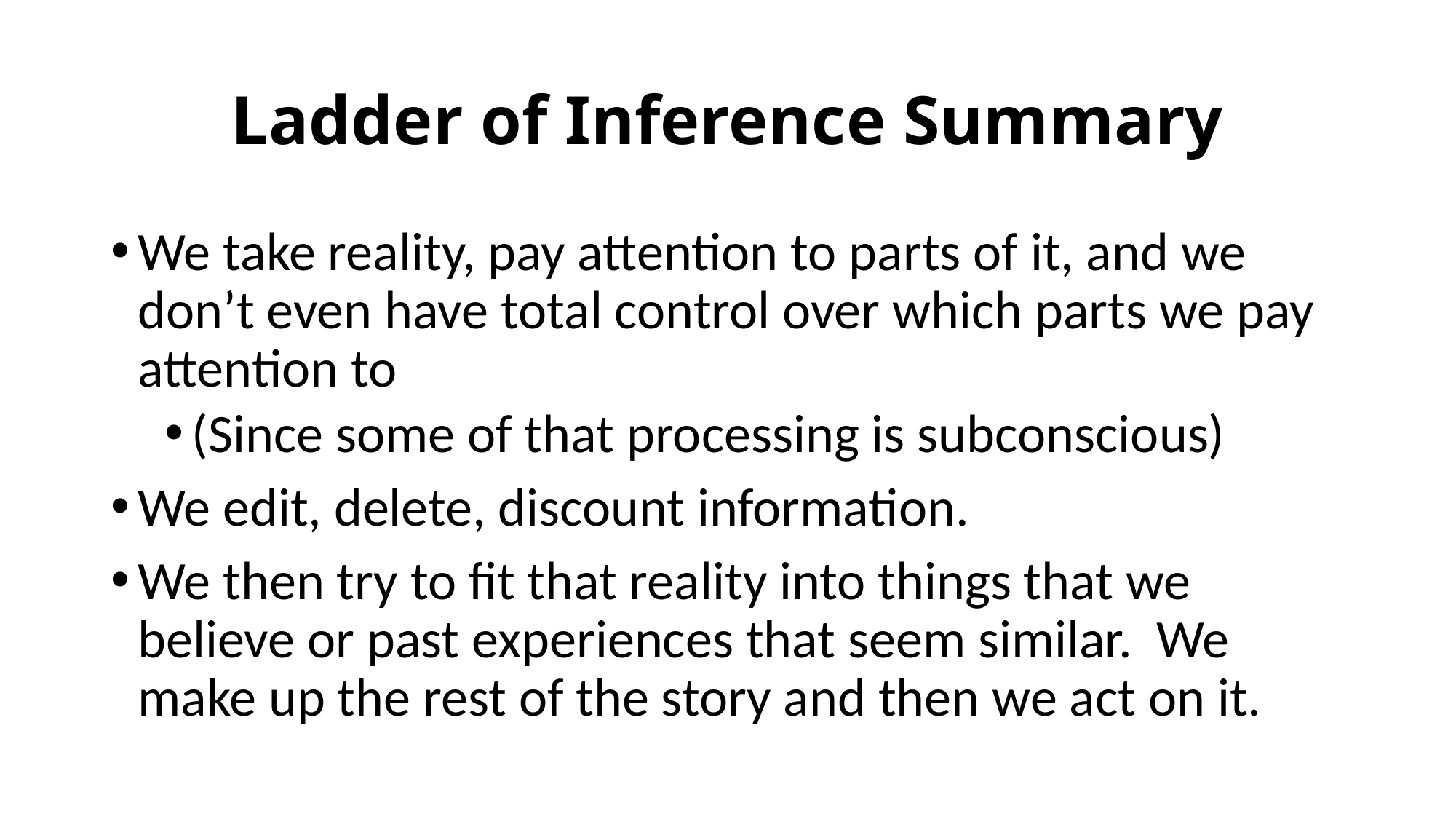

# Ladder of Inference Summary
We take reality, pay attention to parts of it, and we don’t even have total control over which parts we pay attention to
(Since some of that processing is subconscious)
We edit, delete, discount information.
We then try to fit that reality into things that we believe or past experiences that seem similar. We make up the rest of the story and then we act on it.

## Slide 14
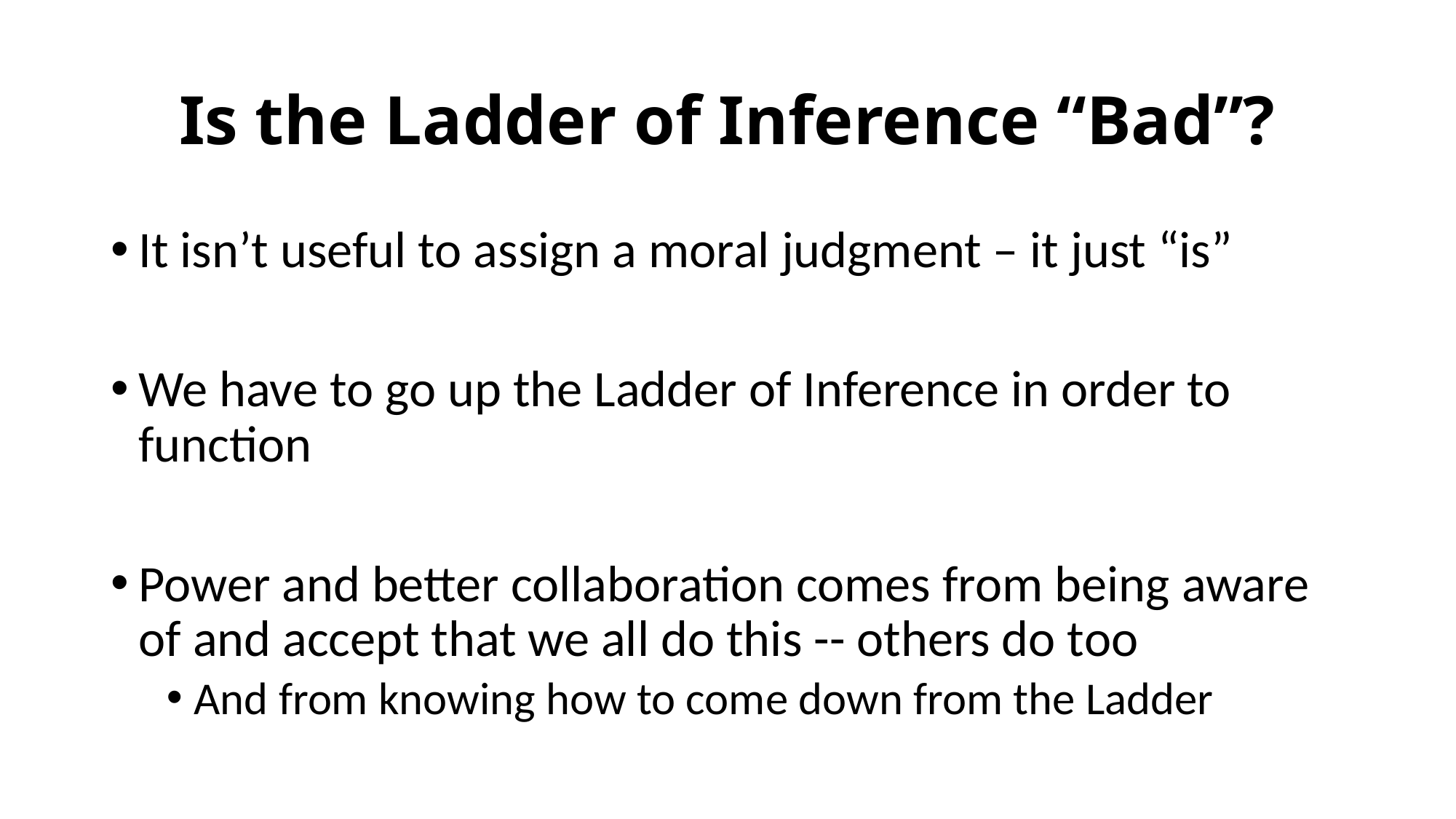

# Is the Ladder of Inference “Bad”?
It isn’t useful to assign a moral judgment – it just “is”
We have to go up the Ladder of Inference in order to function
Power and better collaboration comes from being aware of and accept that we all do this -- others do too
And from knowing how to come down from the Ladder

## Slide 15
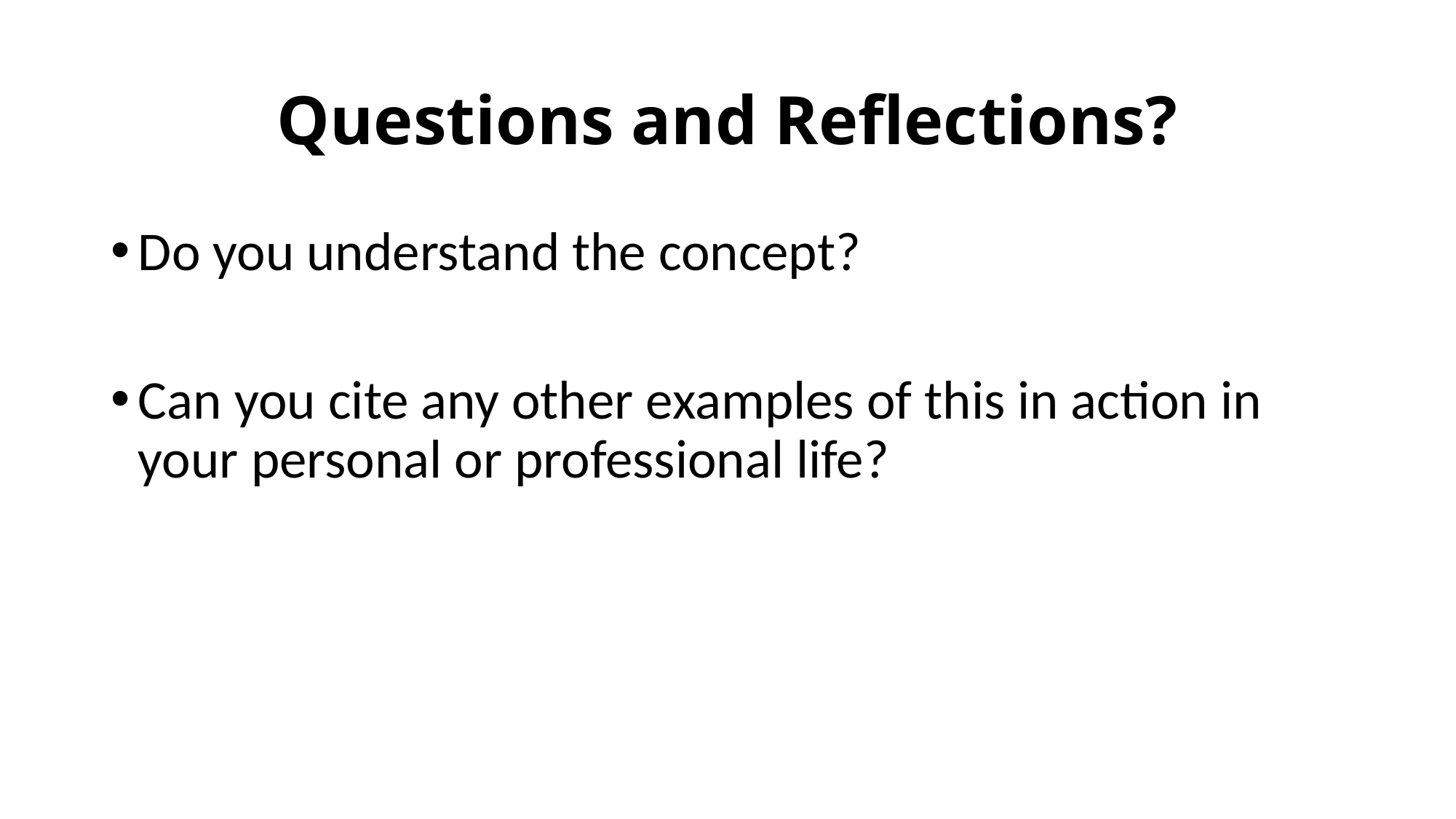

# Questions and Reflections?
Do you understand the concept?
Can you cite any other examples of this in action in your personal or professional life?

## Slide 16
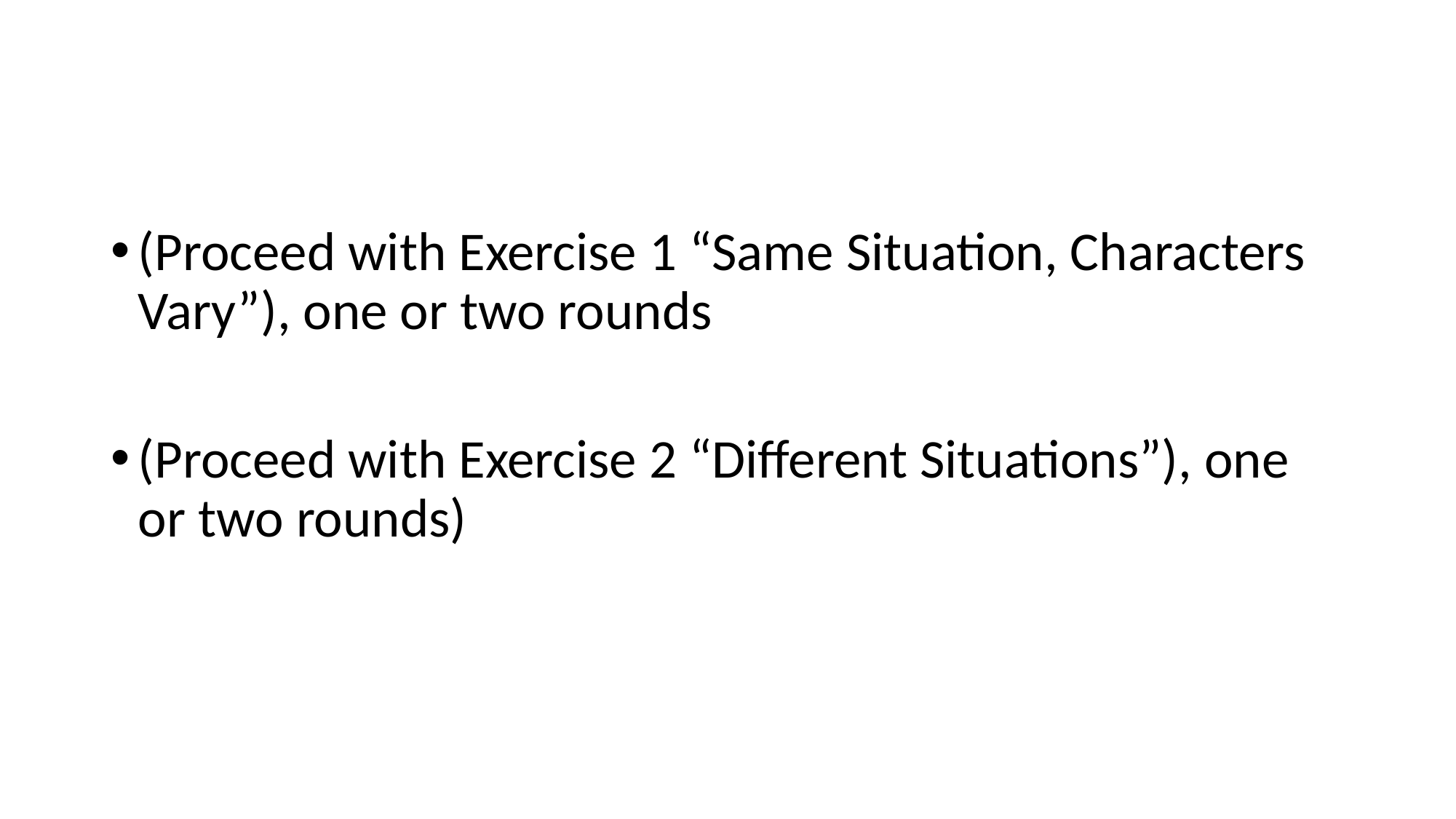

#
(Proceed with Exercise 1 “Same Situation, Characters Vary”), one or two rounds
(Proceed with Exercise 2 “Different Situations”), one or two rounds)

## Slide 17
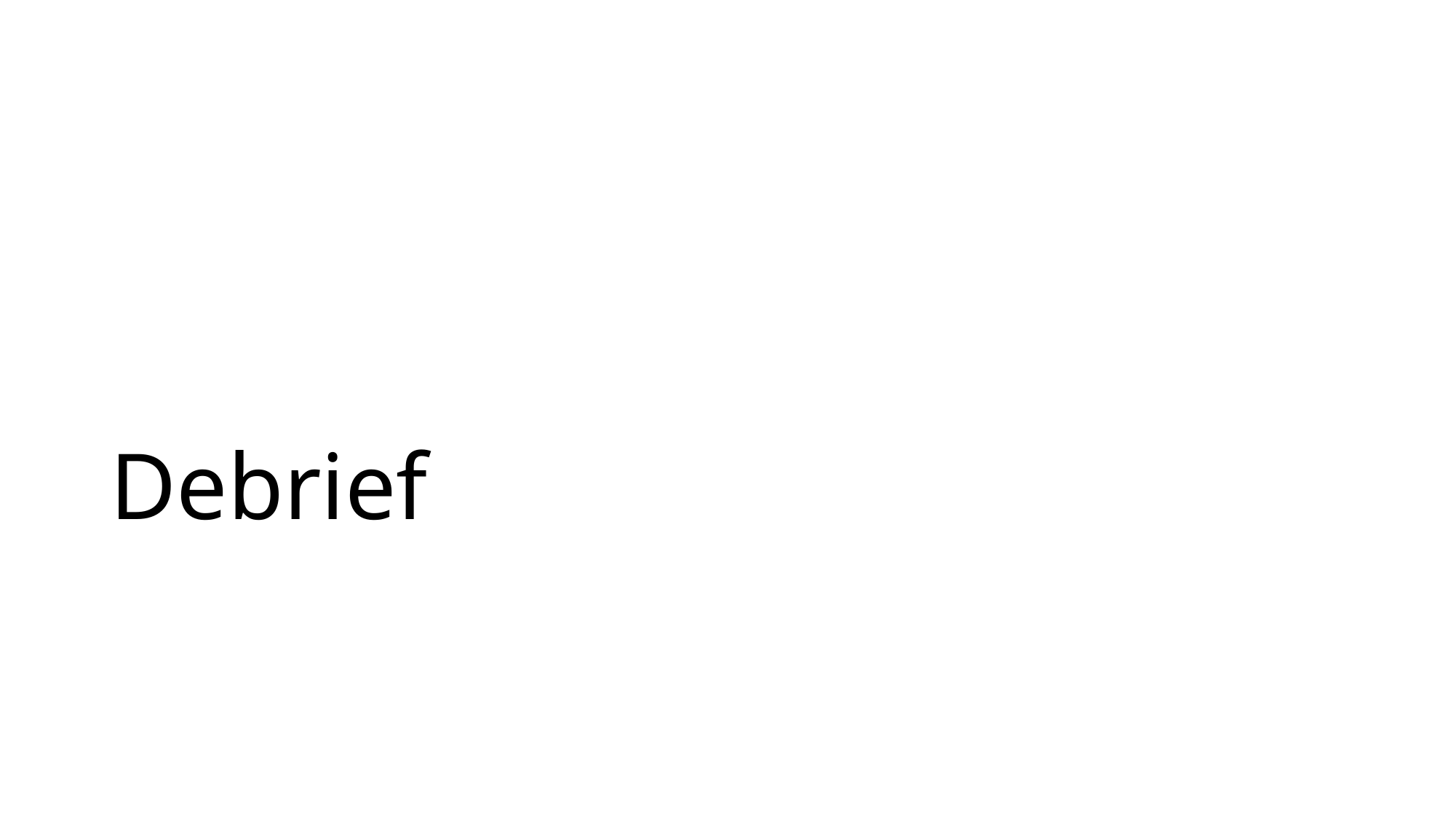

# Debrief

## Slide 18
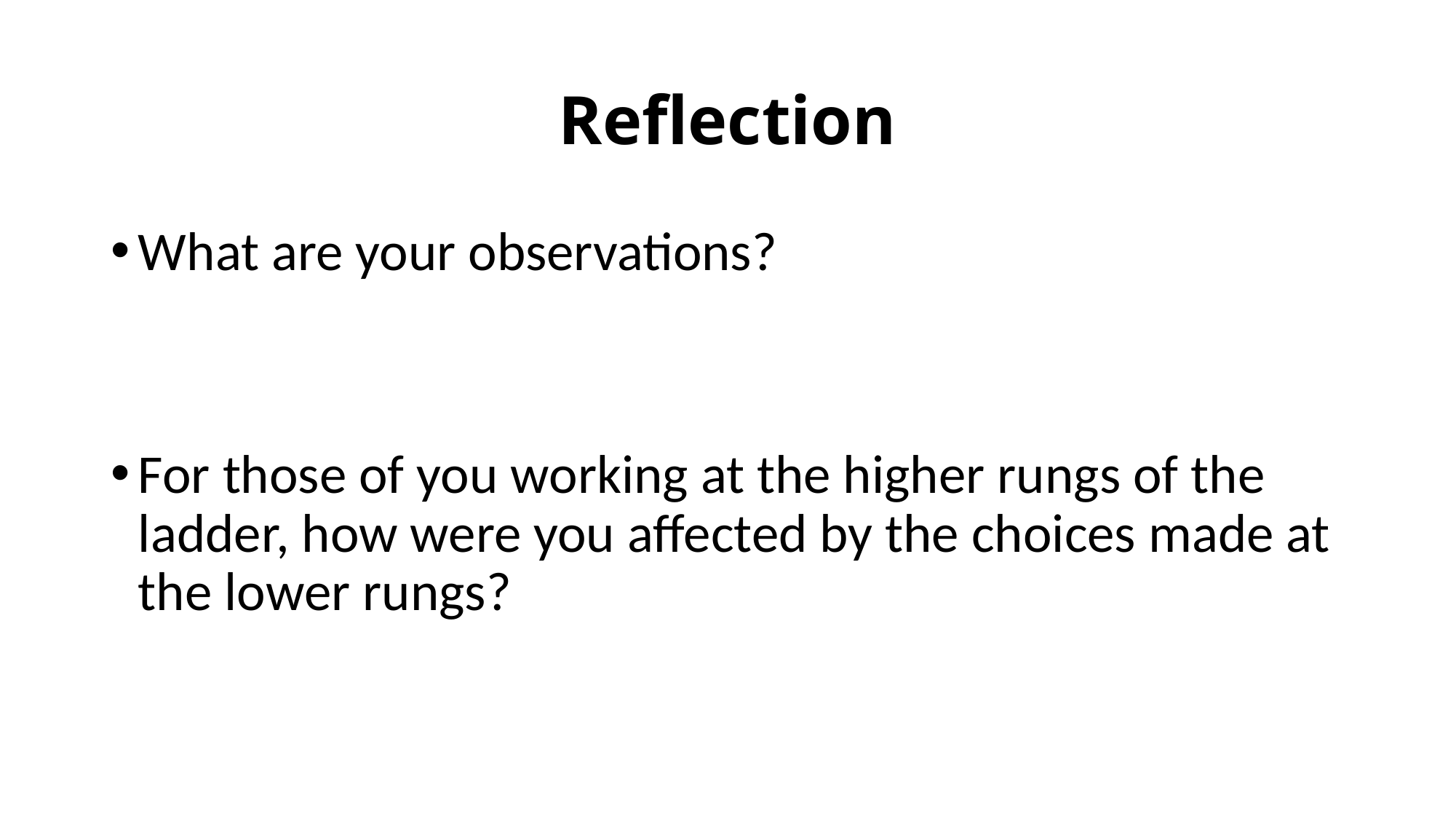

# Reflection
What are your observations?
For those of you working at the higher rungs of the ladder, how were you affected by the choices made at the lower rungs?

## Slide 19
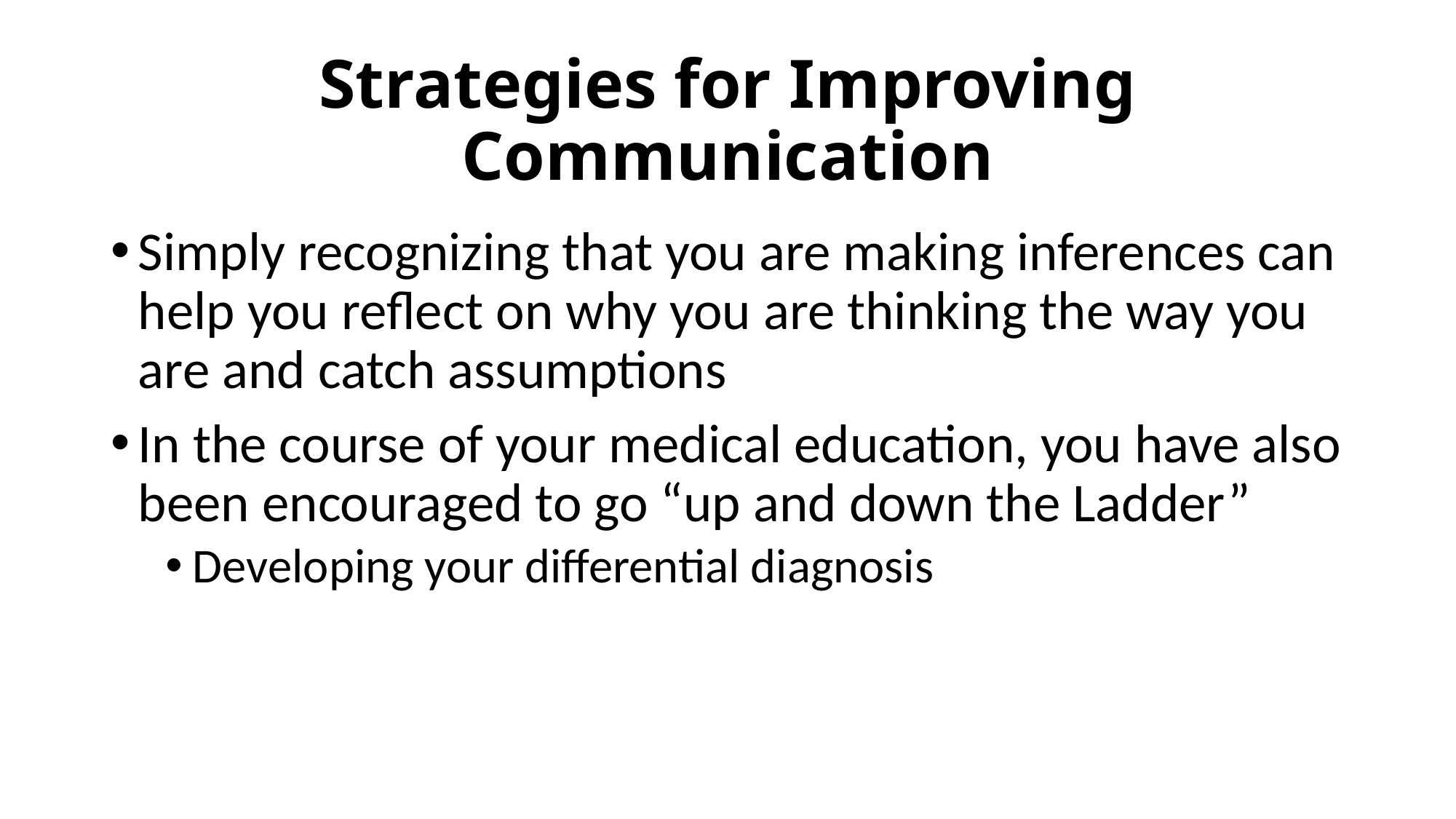

# Strategies for Improving Communication
Simply recognizing that you are making inferences can help you reflect on why you are thinking the way you are and catch assumptions
In the course of your medical education, you have also been encouraged to go “up and down the Ladder”
Developing your differential diagnosis

## Slide 20
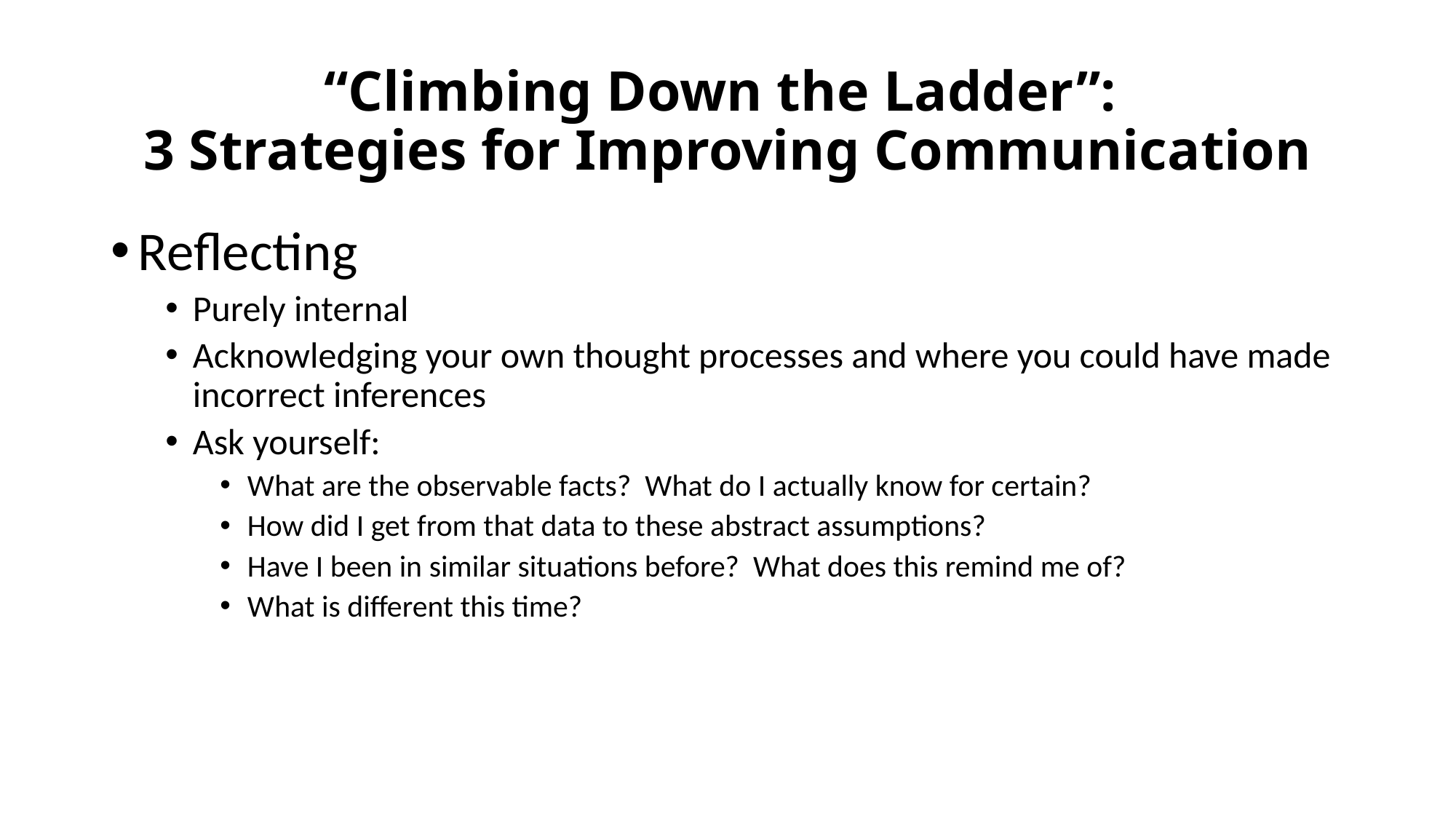

# “Climbing Down the Ladder”: 3 Strategies for Improving Communication
Reflecting
Purely internal
Acknowledging your own thought processes and where you could have made incorrect inferences
Ask yourself:
What are the observable facts? What do I actually know for certain?
How did I get from that data to these abstract assumptions?
Have I been in similar situations before? What does this remind me of?
What is different this time?

## Slide 21
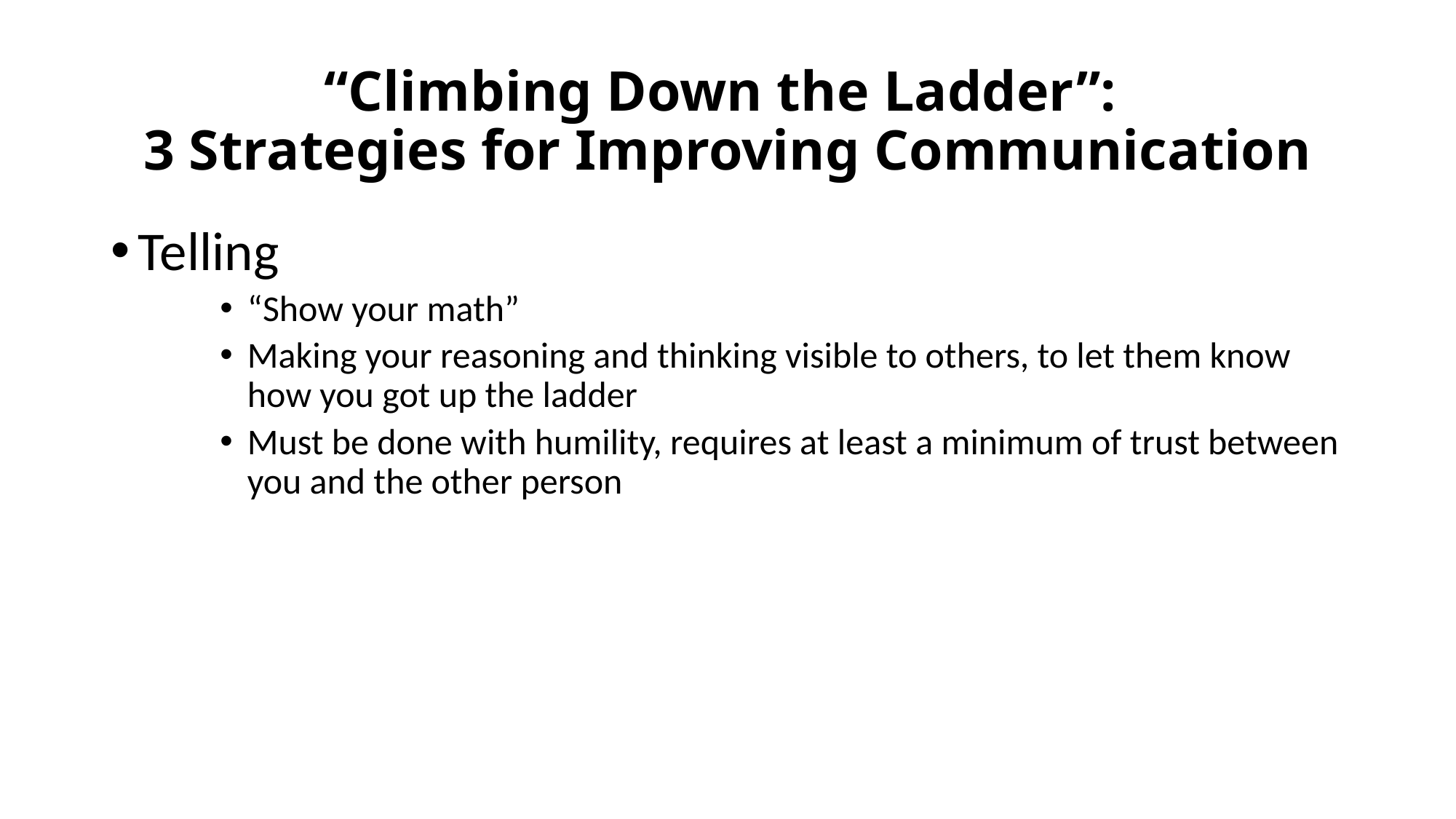

# “Climbing Down the Ladder”: 3 Strategies for Improving Communication
Telling
“Show your math”
Making your reasoning and thinking visible to others, to let them know how you got up the ladder
Must be done with humility, requires at least a minimum of trust between you and the other person

## Slide 22
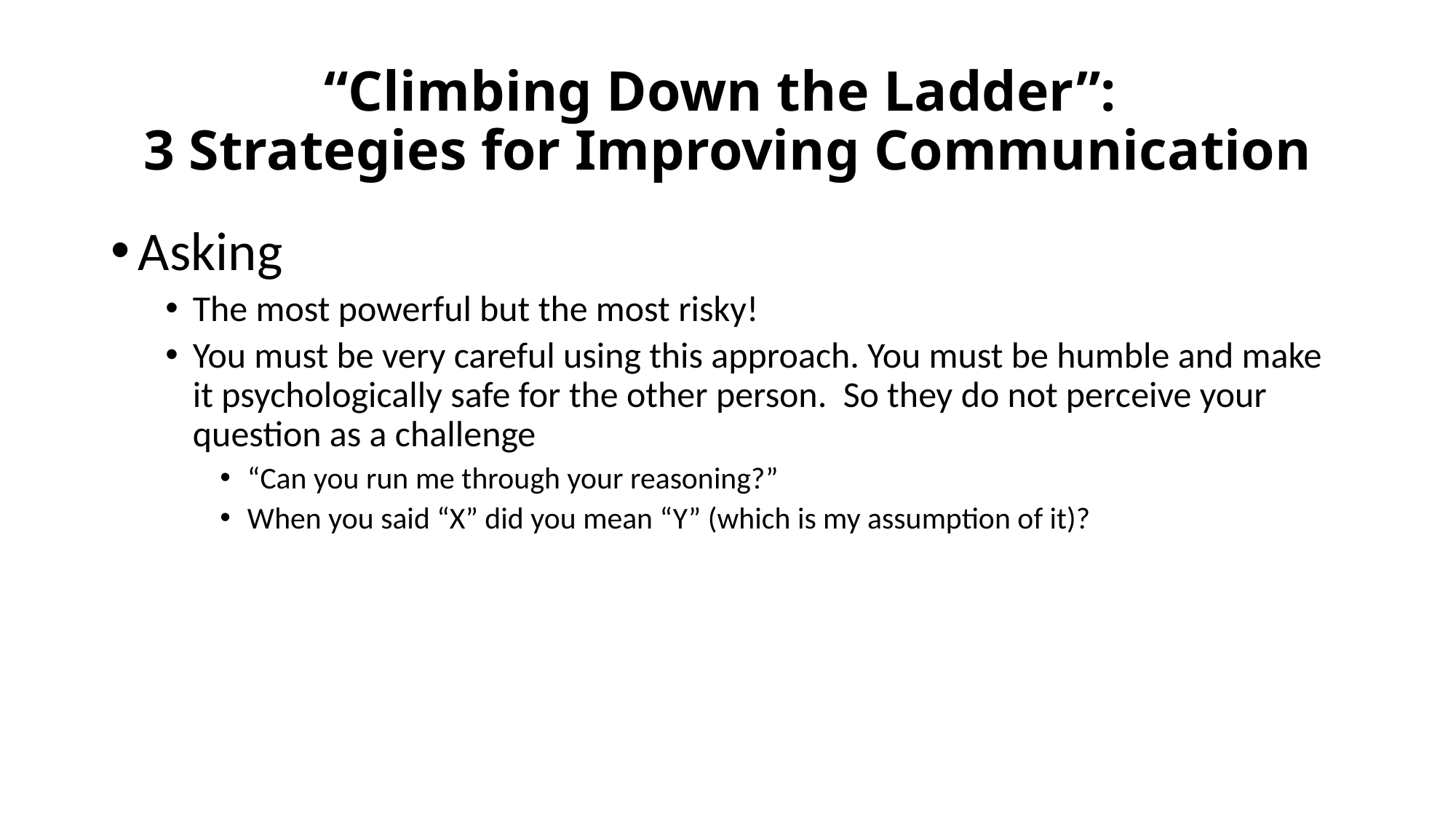

# “Climbing Down the Ladder”: 3 Strategies for Improving Communication
Asking
The most powerful but the most risky!
You must be very careful using this approach. You must be humble and make it psychologically safe for the other person. So they do not perceive your question as a challenge
“Can you run me through your reasoning?”
When you said “X” did you mean “Y” (which is my assumption of it)?

## Slide 23
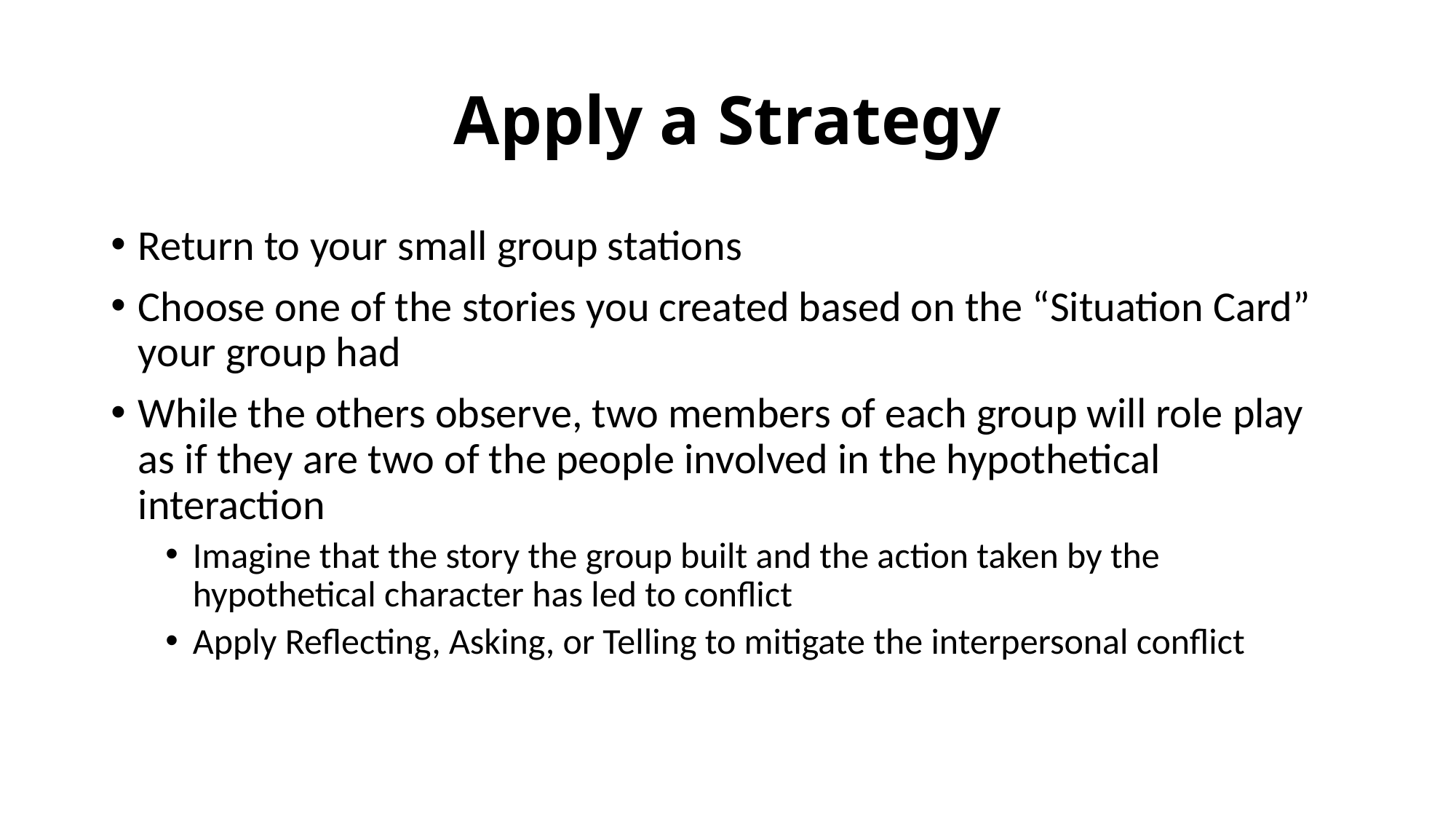

# Apply a Strategy
Return to your small group stations
Choose one of the stories you created based on the “Situation Card” your group had
While the others observe, two members of each group will role play as if they are two of the people involved in the hypothetical interaction
Imagine that the story the group built and the action taken by the hypothetical character has led to conflict
Apply Reflecting, Asking, or Telling to mitigate the interpersonal conflict

## Slide 24
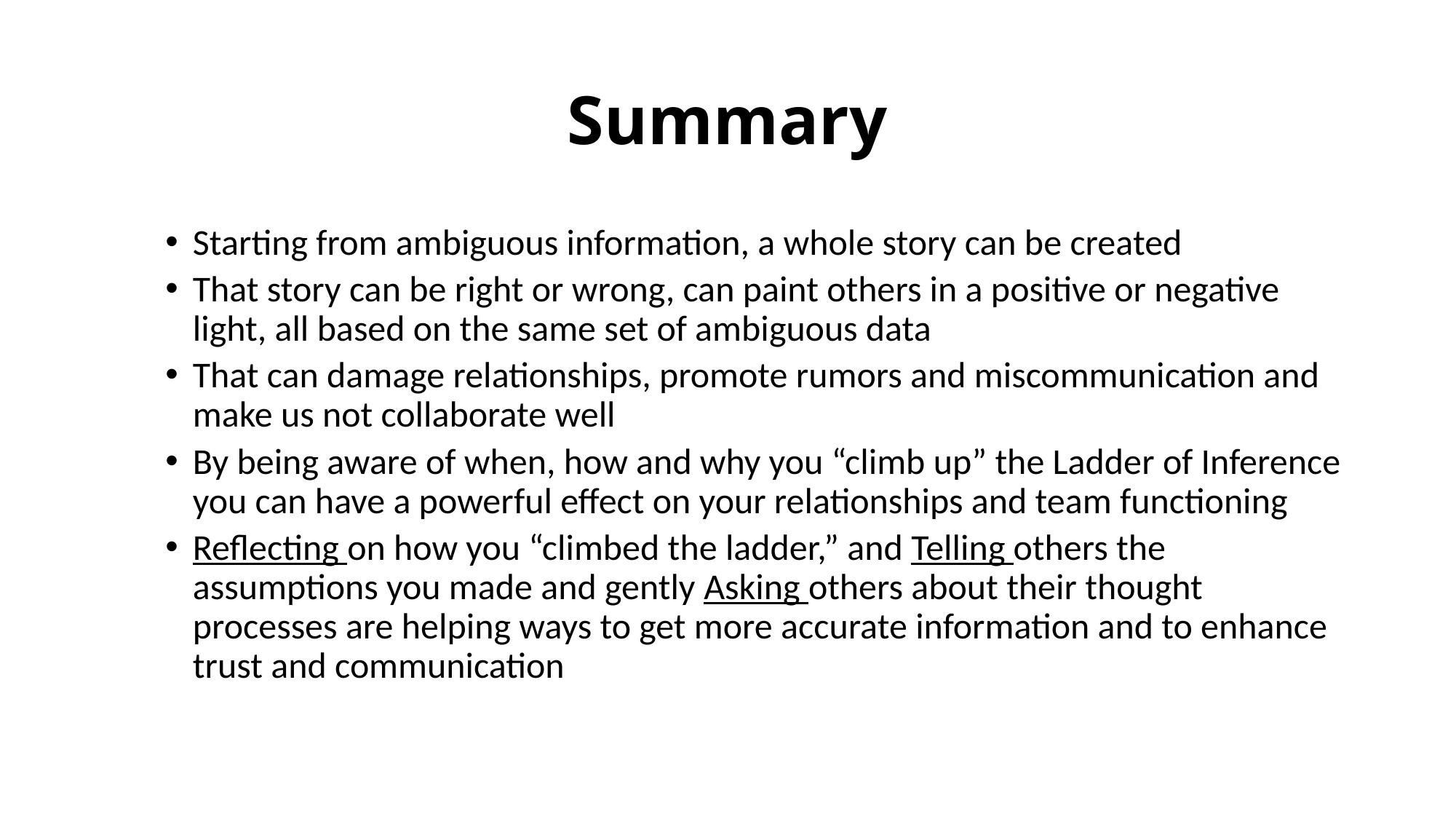

# Summary
Starting from ambiguous information, a whole story can be created
That story can be right or wrong, can paint others in a positive or negative light, all based on the same set of ambiguous data
That can damage relationships, promote rumors and miscommunication and make us not collaborate well
By being aware of when, how and why you “climb up” the Ladder of Inference you can have a powerful effect on your relationships and team functioning
Reflecting on how you “climbed the ladder,” and Telling others the assumptions you made and gently Asking others about their thought processes are helping ways to get more accurate information and to enhance trust and communication

## Slide 25
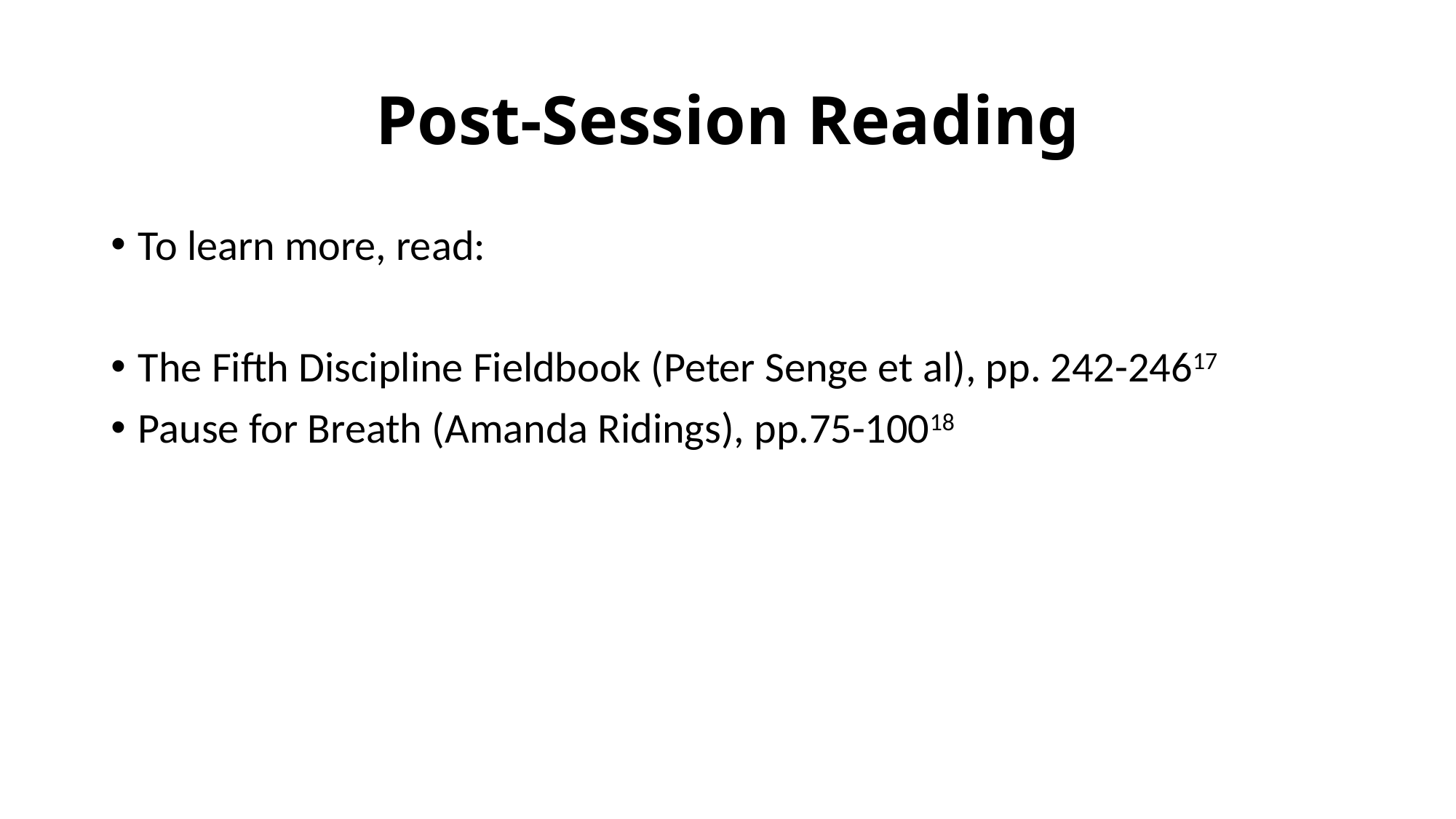

# Post-Session Reading
To learn more, read:
The Fifth Discipline Fieldbook (Peter Senge et al), pp. 242-24617
Pause for Breath (Amanda Ridings), pp.75-10018
