# Supplementary material for: What's Happening in Your Head: Overcoming Our Assumptions to Work Better Together
Source: MedEdPORTAL. 2020 Nov 30;16:11034. doi: 10.15766/mep_2374-8265.11034 (PMC7703482; doi:10.15766/mep_2374-8265.11034)
Supplement: Supplementary file 1 — Ladder of Inference Poster.pptxLadder of Inference Poster.docxCharacter Cards.docxSituation Cards.docxRung Concept Cards.docxLadder of Inference Presentation.pptxExercise 1 Instructions and Talking Points.docxExercise 2 Instructions and Talking Points.docxLadder of Inference Workshop Assessment Tool.docx [file mep_2374-8265.11034-s001.zip › G. Exercise 1 Instructions and Talking Points.docx]

**Appendix G. Exercise 1 Instructions and Talking Points**

The facilitator sorts the participants into groups of 3-6 participants. This can be done by “counting off” participants while in the circle, with the facilitator ensuring a mix of faculty and residents into each group. Fewer members per group is preferred, but because each group will receive a unique “Character Card” having more groups requires development of additional cards.

The facilitator should ensure that one set of Rung Cards (Appendix E) have been placed at each station for every round that will be done. If using masking tape instead of picturing hanging strips, one roll should be placed at each station to attach the Rung Cards to the poster.

1. Facilitator tells the group:
   1. “We are going to do some exercises to get you used to applying the concepts from the Ladder of Inference. There are 7 rungs on the Ladder of Inference. But the way this is designed, we only need to be responsible for the upper 5 concepts. Each team member should choose to be responsible for a concept on a rung of the ladder. Take a minute now to decide who will be responsible for which concept. If there are not 5 people in a group then it is OK for someone to take on more than one concept. Decide now…. Each person should take a blank card for every Rung Concept for which they are responsible. (Alternatively, each group may collaborate to fill in all Rung Concept Cards)
   2. “Your group is going to pretend to be inside the mind of one imaginary person. You are going to be put into a situation which I’ll tell you about in a moment. Your job is going to be to imagine how that imaginary person could “climb up the ladder” in a certain situation. Your group will then report out to the larger group how you went “up the Ladder.”
   3. “First, you’ll need to get to know your imaginary person. I’m going to hand out a card that describes your group’s imaginary person. Each group will have a different card. When I hand it to you, share it with the rest of your group.”
      1. The Facilitator randomly hands out one unique “Character Card” for each group (Appendix C). It is not necessary but can be appealing to match the color of the Character Card to the group’s Rung Cards. This can be but does not have to be color-coded though it is nice to match the “Character Card” to the group’s colored Rung Cards
   4. “You now know your group’s character. I’ll tell you the situation in a moment. Here’s how we’ll proceed once I tell you the situation. What I am going to tell you is the Reality and Facts. Because I can’t really describe every aspect of reality, that means what I will tell you is also Selected Reality, just the slice of reality you are being told to pay attention to in this scenario. So those lower two rungs are ‘given’ by the scenario I’m about to tell you. But the rest of the rungs need to be filled in by the group. Starting with the person responsible for “Interpreted Reality,” that person’s job is to imagine what is going on in the head of the character. How do they interpret the reality that they have just been told about? Keep this very short and basic, you’re not telling the whole story yet when you are interpreting reality. That person can work with the rest of the group if they want to, but they need to write down on their card how they are interpreting reality.”
   5. “Once they have written this down, they tape/attach it to the corresponding rung on the image of the Ladder of Inference poster for their group.
   6. “When the Interpreted Reality person is finished, the person responsible for “Assumptions” writes down their assumptions based on Interpreted Reality. And so forth up until you post an “Action” at the highest rung.”
   7. “Any questions about the process? You know your character. Here is the scenario:
      1. Imagine you are a resident just finishing up preparing your notes on your laptop before starting inpatient walking rounds. In that last second as you close your laptop’s cover and won’t have a chance to look at email for the next two hours, an email from the Program Director pops up and reads “Be in my office at 1:30 this afternoon”
   8. “That’s the scenario. And that fills in Reality and Selected Reality. Now start imagining how your imaginary resident interprets reality and post it on your ladder.” The next person adds assumptions they make.
   9. When this is concluded in 10-15 minutes, the Leader asks each group to appoint a spokesperson to share with the larger group how they climbed the Ladder, starting with Interpreted Reality. “Please describe your character by reading the Character Card. Then starting with Interpreted Reality, say the level of the rung you are on and then say what you wrote down, working your way up the Ladder.”
   10. After the final group reports, the Leader asks:
       1. “Did you notice any themes or patterns?”
       2. “That was an intentionally ambiguous Scenario. Was there a different way that situation could have gone, that you could have interpreted it?”
   11. “Remove what you posted to get ready for the next version.”
   12. If you have time and have used up half or fewer of your Character Cards you may do another round of this or proceed to the next Exercise

Facilitator Notes:

- There are no “right answers” to the stories participants create -- to the inferences they draw. The beliefs adopted by and actions taken by each group’s hypothetical character can be realistic or outlandish. Your role is to ensure the small groups follow the workshop process properly, to help them walk through and thus become explicitly aware of how we make inferences.
- Encourage creativity. Teams have a lot of fun with creating the stories and reporting them to the group. This is usually gets the participants very highly engaged and builds a memorable experience.
- Sense whether there is tension and emphasize that this exercise is for a hypothetical residency, not necessarily this residency
- Expect that the stories generated often involve the hypothetical characters making inferences that are negative -- that they are going to be in trouble with the Program Director.
