# Supplementary material for: What's Happening in Your Head: Overcoming Our Assumptions to Work Better Together
Source: MedEdPORTAL. 2020 Nov 30;16:11034. doi: 10.15766/mep_2374-8265.11034 (PMC7703482; doi:10.15766/mep_2374-8265.11034)
Supplement: Supplementary file 1 — Ladder of Inference Poster.pptxLadder of Inference Poster.docxCharacter Cards.docxSituation Cards.docxRung Concept Cards.docxLadder of Inference Presentation.pptxExercise 1 Instructions and Talking Points.docxExercise 2 Instructions and Talking Points.docxLadder of Inference Workshop Assessment Tool.docx [file mep_2374-8265.11034-s001.zip › H. Exercise 2 Instructions and Talking Points.docx]

**Appendix H. Exercise 2 Instructions and Talking Points**

Following Exercise 1, participants remain at their stations in the same groups. Another set of Rung Cards (Appendix E) is given to each group for this round.

1. Facilitator tells the group:
   1. “We’re going to do a slightly different version of the exercise. In this version, each group will have its own different situation. The character isn’t as important now. It can be anyone real or imagined or a collective of the group imagined as a single person.”
   2. “We’re going to do the same exercise and I will give each group its own ‘Reality and Facts’ and ’Selected Reality.’ Once I give you that “Situation Card,” go ahead and begin working your way up the ladder just as you did before. When the Interpreted Reality person is finished, the person responsible for “Assumptions” writes down their assumptions based on Interpreted Reality. And so forth up until you post an “Action” at the highest rung.”
      1. The Leader hands out a “Situation Card” for each group (Appendix D). This can be but does not have to be color-coded though it is appealing to match the “Situation Card” to the group’s colored Rung Cards
   3. The group is instructed to proceed writing on the Rung Cards and attaching them to the posters as in Exercise 1.
   4. When this is concluded in 10-15 minutes, the Leader asks each group to appoint a spokesperson to share with the larger group how they climbed the Ladder, starting with Interpreted Reality. “Please describe your character by reading the Character Card. Then starting with Interpreted Reality, say the level of the rung you are on and then say what you wrote down, working your way up the Ladder.”
   5. After each group reports, the Leader asks:
      1. “That was an intentionally ambiguous Situation. Was there a different way that situation could have gone, that you could have interpreted it?”

Facilitator Notes:

- There are no “right answers” to the stories participants create -- to the inferences they draw. The beliefs adopted by and actions taken by each group’s hypothetical character can be realistic or outlandish. Your role is to ensure the small groups follow the workshop process properly, to help them walk through and thus become explicitly aware of how we make inferences.
- Encourage creativity. Teams have a lot of fun with creating the stories and reporting them to the group. This is usually gets the participants very highly engaged and builds a memorable experience.
