# Supplementary material for: What's Happening in Your Head: Overcoming Our Assumptions to Work Better Together
Source: MedEdPORTAL. 2020 Nov 30;16:11034. doi: 10.15766/mep_2374-8265.11034 (PMC7703482; doi:10.15766/mep_2374-8265.11034)
Supplement: Supplementary file 1 — Ladder of Inference Poster.pptxLadder of Inference Poster.docxCharacter Cards.docxSituation Cards.docxRung Concept Cards.docxLadder of Inference Presentation.pptxExercise 1 Instructions and Talking Points.docxExercise 2 Instructions and Talking Points.docxLadder of Inference Workshop Assessment Tool.docx [file mep_2374-8265.11034-s001.zip › I. Ladder of Inference Workshop Assessment Tool.docx]

# Ladder of Inference Workshop Evaluation

# The following items ask for your impressions of the Ladder of Inference workshop. Please be candid, remembering that you cannot be identified based on your responses to this questionnaire.

Respond to each of the following items on the scale ranging from 1 = *Strongly Disagree* to 7 = *Strongly Agree*.

|  | Strongly Disagree | Strongly  Agree |
| --- | --- | --- |
| 1. This workshop captured and held my interest. | 1 2 3 4 5 6 7 | |
| 2. This workshop was effective in helping me to understand the concept of the Ladder of Inference -- how people make assumptions based on incomplete information.  3. This workshop provided me with a means to understand the dynamics within my group as they relate to making assumptions. | 1 2 3 4 5 6 7  1 2 3 4 5 6 7 | |
| 4. This workshop was effective in making me explicitly aware of the detrimental impact that incomplete or incorrect information can have on a group.  5. This workshop was effective in helping me identify means to mitigate any detrimental impact that assumption-making can have on a group. | 1 2 3 4 5 6 7    1 2 3 4 5 6 7 | |
|  |  | |
| 6. I was able to better identify and be aware of the detrimental impact that making inaccurate or incomplete assumptions can have on interpersonal and team interactions.  7. I was able to identify techniques to minimize the problems caused by inaccurate or incomplete assumptions.  8. I plan to actively apply techniques to minimize the problems caused by inaccurate or incomplete assumptions. | 1 2 3 4 5 6 7  1 2 3 4 5 6 7  1 2 3 4 5 6 7 | |
| 9. This workshop activity would be valuable for future residency classes in our organization. | 1 2 3 4 5 6 7 | |
| 10. This workshop activity would have value in other organizations like ours in the future. | 1 2 3 4 5 6 7 | |

11. On a scale from 1 to 7, please indicate your overall level of satisfaction with this workshop.

Very dissatisfied 1 2 3 4 5 6 7 Very satisfied

12. Year in Residency ____

13. Age ____

14. Gender

__Female

__Male

__Other: __________

__Prefer not to answer

15. Ethnicity

__Hispanic or Latino

__NOT Hispanic of Latino

__Unknown/Not Reported

16. Race

__American Indian or Alaska Native

__Asian

__Black or African American

__Native Hawaiian or Other Pacific Islander

__White

__More than one race

__Unknown/Not Reported

Please provide any additional comments or suggestions about the workshop:
