# Supplementary material for: What's Happening in Your Head: Overcoming Our Assumptions to Work Better Together
Source: MedEdPORTAL. 2020 Nov 30;16:11034. doi: 10.15766/mep_2374-8265.11034 (PMC7703482; doi:10.15766/mep_2374-8265.11034)
Supplement: Supplementary file 1 — Ladder of Inference Poster.pptxLadder of Inference Poster.docxCharacter Cards.docxSituation Cards.docxRung Concept Cards.docxLadder of Inference Presentation.pptxExercise 1 Instructions and Talking Points.docxExercise 2 Instructions and Talking Points.docxLadder of Inference Workshop Assessment Tool.docx [file mep_2374-8265.11034-s001.zip › _Educational_Summary_Report_11034.pdf]

MedEdPORTAL® | The AAMC Journal of Teaching and Learning Resources

Original Publication Open Access

What's Happening in Your Head: Overcoming Our Assumptions to Work Better Together

Daniel Schlegel, MD, MHA*, Jessica Parascando, MPH

*Corresponding author: dschlegel@pennstatehealth.psu.edu

Abstract

Introduction: It is crucial that residents learn in environments that are psychologically safe and free of morale-harming rumors. This workshop introduced the Ladder of Inference as a means for programs to foster psychological safety, mitigate against rumors, and increase trust. **Methods:** Residents and faculty of two residency programs (cohorts A and B) were introduced to the Ladder of Inference. After an interactive discussion, small groups applied the concept to engaging, highly relevant hypothetical situations. Debriefing and reflection followed the group work. Finally, attendees completed an assessment of the workshop's effectiveness (10 7-point questions) and their satisfaction (one 7-point question). **Results:** Seventeen residents from cohort A completed the workshop, and 15 completed the assessment (response rate: 88%). Participants found it favorable ($M = 65.9$ out of 70) and satisfactory (6.3 out of 7). Twenty-eight residents and faculty from cohort B completed the workshop, and 15 completed the assessment (response rate: 54%). Cohort B participants also found the workshop favorable ($M = 64.8$) and satisfactory (6.8). Both cohorts felt the workshop was effective in helping them understand the concept of the Ladder of Inference (6.5 for both cohorts) and would provide value in their residency program in the future (6.1 for cohort A, 6.7 for cohort B) and at other organizations (6.3 for cohort A, 6.7 for cohort B). **Discussion:** This workshop is an effective method whereby participants can develop and apply an understanding of the Ladder of Inference. This shared understanding can promote trust and enhance the resilience of a program.

Keywords
Morale, Trust, Interpersonal Communication, Rumor, Workshop, Beliefs, Ladder of Inference, Communication Skills, Professionalism, Games

Educational Objectives

By the end of the workshop, participants will be able to:

1. Describe a mental framework called the Ladder of Inference whereby people develop beliefs and take actions based on incomplete and incorrect information.
2. Apply their understanding of that framework to the program, organization, or community of which they are a member.
3. Identify the detrimental impact that incomplete and incorrect information can have on the program, organization, or community of which they are a member.
4. Practice or observe reflecting, asking, and telling as techniques whereby the detrimental impact of that incomplete and incorrect information on the program, organization, or community of which they are a member can be mitigated.

Citation:
Schlegel D, Parascando J. What's happening in your head: overcoming our assumptions to work better together. *MedEdPORTAL*. 2020;16:11034.
https://doi.org/10.15766/mep_2374-8265.11034

Introduction

Psychological safety has been identified as a key factor in the success of organizations that are complex and require expertise and close collaboration.[1] The idea that it is safe to fail makes individuals willing to "employ or express themselves physically, cognitively, and emotionally during role performances" rather than "withdraw and defend their personal selves."[2] When individuals feel psychologically safe, they can overcome the defensiveness or anxiety they may feel when presented with data contradicting their expectations or hopes. Then, they are free to focus on collective goals, prevention, and improvement rather than on self-protection.[3]

Copyright © 2020 Schlegel and Parascando. This is an open-access publication distributed under the terms of the Creative Commons Attribution license.

In a residency program, learners are under pressure to acquire and develop new skills unerringly while under the intense scrutiny of faculty supervisors. In addition, high-achieving young physicians have high expectations of themselves, making them particularly vulnerable to challenges to their psychological safety. When they receive negative feedback, they may be at particular risk of perceiving threats to their self-image, compromising their ability and desire to enhance their own performance and the collective performance of their residency. It has been suggested that learners' desire to protect their self-image is tied to a desire to avoid internal shame. In turn, low psychological safety may thus have negative effects on learning and well-being.[4]

Psychological safety can have positive effects on patient care and is predictive of residents' intention to report adverse events.[5] Psychological safety has been shown be an important factor in resident satisfaction in the clinical learning environment. The Accreditation Council for Graduate Medical Education (ACGME) Annual Survey of Residents is an anonymous, externally administered measure assessing trainees' satisfaction with their learning environment.[6] Results from recent years (2016-2019) suggest that there is room for improvement nationally on two items that could be potential markers for psychological safety: "Satisfied with process to deal with problems and concerns" and "Residents can raise concerns without fear." The ACGME does not provide a range of scores. However, leaders of programs scoring low on these measures may be particularly motivated to improve the psychological safety of their programs.

In the clinical environments of 23 neonatal intensive care units, psychological safety has been shown to predict success for teams learning and implementing new practices, in turn demonstrating lower risk-adjusted mortality.[7,8]

Aside from the stress associated with low psychological safety, an additional potential stressor for residency programs is rumors. The charged environment and long hours spent together can foster camaraderie but are also fertile for promoting rumors and gossip between and among residents and the faculty they work alongside.[9]

A residency program's faculty and program director are simultaneously teachers of residents and representatives of their mutual employer. As educators, the faculty must foster a safe and accessible educational environment in accord with ACGME guidelines. As representatives of the employer, the faculty must manage the program in a coherent, rational way in accordance with institutional requirements and legal regulations. At times, there may be tension between adhering to those requirements and fostering a healthy educational environment. For example, limitations on information sharing, such as may be necessary during a sudden departure of a member of the residency, can promote distrust among members of the program. Particularly in such times of crisis and in the context of a high-pressure residency environment, rumors can sprout freely and have a profound and negative impact on trust, morale, and perceived psychological safety within the educational community.

Rumor theory explains that rumors tend to spring from the collective concerns of a group of interconnected individuals.[10] Rumors are propositions of allegations without corroborative evidence, and they "scamper about organizations like some mischievous poltergeist, until skillful managers exorcise the allegations or the allegations vanish into thin air."[10] They exist to fill in information gaps as a form of speculation when not enough information is available.[10] In a residency program, change inevitably occurs within a network of highly interconnected residents and faculty who share overlapping interests. When legal or other reasons mandate that information cannot be fully shared to fill in the blanks during times of change, the information famine can readily spawn such a poltergeist.

Rahmani recently expressed skepticism that program directors are equipped to manage rumors within their programs and proposed a framework for rumor management.[11] The four steps proposed are to (1) remain indifferent to avoid empowering the rumor, (2) understand how the impact will affect various stakeholders, (3) measure the pros and cons of addressing versus ignoring the rumor, and (4) remedy and redress the rumor through the proper route, possibly with assistance from Human Resources or the Office of Graduate Medical Education. Other authors within the health care domain suggest means of stopping rumors. Ribeiro and Blakeley recommend assessing and analyzing the grapevine, identifying those individuals who are habitual spreaders of rumors, and keeping employees informed.[12] Chase and Stuart suggest preventing and influencing rumors, debriefing and transforming them, and clarifying and informing.[13] These approaches generally depend on a person with authority presenting facts to dispel the rumor. Yet these strategies of keeping employees informed and presenting facts to dispel the rumor are not available when an information blackout must be imposed. In fact, if a leader shares information with residents and faculty too widely, it can result in charges of defamation that can be considered a tort under state laws.[14]

How, then, can a program director or other organizational leader maintain an environment of trust and reduce anxiety in the

Copyright © 2020 Schlegel and Parascando. This is an open-access publication distributed under the terms of the Creative Commons Attribution license.

context of uncomfortable, possibly unwelcome change about which little information can be shared?

The Ladder of Inference, as described by Argyris, suggests a means whereby organization leaders such as program directors can treat or even inoculate against rumors that could be detrimental to harmonious program functioning.[15] The ladder is a mental model that acknowledges the shortcuts people take in their thinking and the erroneous conclusions that can result. The lowest rung of the metaphorical ladder is observable data and experiences—things that concretely occur in the physical world. Given humans' finite capacity to absorb and attend to such data, only a portion of what occurs in the world receives attention. What is attended to may be informed by past experiences. Receivers of the data assign meaning to that slice of reality, then fill in with their assumptions the vast amount of relevant information not observed. They make conclusions about the information, become certain of their belief, and take action based upon it. Between the time that observable data from the external world are perceived and action is ultimately taken, an enormous amount of mental processing manipulates and massages reality within the brain's black box, all in the span of milliseconds. When there is a partner or group of partners in a team interaction, the possibilities for misunderstanding are abundant as the participants all race up their own ladders of inference. Imperfect as this process may be, it is a consequence of humans' limited capacity to process information and is thus a ubiquitous feature of workplace interactions.

The Ladder of Inference has been suggested among health care managers as a tool to facilitate candid conversations, to decrease errors and serious sentinel events, and to protect against staff dissatisfaction and turnover.[16]

Residents are immersed in a training environment that can pose a continuous threat to their psychological safety and harm their morale. The social milieu of a residency is fertile ground for the generation or propagation of rumors, which can also harm morale.

We believe that previously proposed approaches for program directors to manage rumors are inadequate, especially when divulging information is not an option. We also believe that opportunities exist to create greater psychological safety in residency programs. For a target audience of residency program faculty and residents, our goal was to utilize the Ladder of Inference concept to facilitate trustful communication in order to mitigate against damaging rumors and enhance psychological safety in general.

We introduced the concept of the Ladder of Inference as a means of coming to a shared understanding about how all members of a residency habitually and by necessity climb up this ladder. With the shared acknowledgment that team members inevitably make assumptions and draw incorrect conclusions, we were able to introduce a tool that could inoculate a program against rumors, create a resilient and trusting team, and enhance psychological safety even in challenging situations and information famines. This novel application of a model from systems theory in the medical education environment has widespread applicability throughout educational, clinical, and organizational settings.

The intended audience for this activity is the residents, faculty, and director of a singular residency program. Appropriate settings include a program retreat or a dedicated residency half-day didactic or conference session.

Methods

Workshop

The workshop was implemented on two occasions with different participants. For cohort A, we presented the workshop as a 2-hour activity within the context of regularly scheduled Thursday morning didactics for the residents of a mid-Atlantic psychiatry residency program based within an academic medical center. The group included 15 of the program's 17 residents as well as the program director and one faculty member. Participants needed no prerequisite knowledge, although the facilitator had to have familiarity with the presented concepts and the proper operation of the activity. The facilitator was a former director of a family medicine residency at the same institution, as well as being an investigator in the study. The participants of cohort B were the 24 residents and four faculty of a mid-Atlantic family medicine residency program at a different hospital affiliated with a different academic medical center. Cohort B received a 3-hour version of the workshop with the same facilitator as cohort A.

Preparation

Prior to the workshop, the facilitator had to be familiar with the Ladder of Inference concept from reading *The Fifth Discipline Fieldbook*[17] and/or *Pause for Breath.*[18]

After identifying the participants, we calculated the number of small groups for the activity such that each group ideally contained three to four participants but no more than six. Each group had one clipboard and one permanent marker. We printed one large poster (30 inches horizontal × 60 inches vertical) of the Ladder of Inference (Appendix A or B) for each small group. Ample masking tape, thumbtacks, or two-sided, removable

Copyright © 2020 Schlegel and Parascando. This is an open-access publication distributed under the terms of the Creative Commons Attribution license.

adhesive picture-hanging strips (preferred) were available to affix cards to posters and posters to the wall.

The workshop required a room large enough to accommodate all the participants seated in a circle as well as breakout space for smaller groups. Ideally, this was a large, open room with ample chairs, few tables or other obstructions, and plentiful wall space upon which posters could be affixed.

In advance of the activity, we printed one unique Character Card from Appendix C on colored cardstock for each small group. One unique Situation Card from Appendix D was similarly printed for each small group. The sample cards in Appendices C and D could be used as they were or modified to suit the identity of the participants. Additional cards could be developed if the number of small groups exceeded the number of cards provided. If less than half the Character or Situation Cards were used, additional rounds of the exercises could be run with the unused cards at the discretion of the facilitator. For each round, we also printed one set of Rung Concept Cards (Appendix E) matching the color of a group's Character and Situation Cards for each small group.

On the day of the activity, stations for each small group were arranged throughout the room with adequate space between them. At each station were a clipboard and permanent marker, and a copy of the Ladder of Inference poster hung on the wall or sat upon an easel.

At the outset of the workshop, participants' chairs were arranged in a circle. The facilitator proceeded with the presentation using the prominently displayed Ladder of Inference poster or a screen image.

The first half of Appendix F (slides 1-15) presented a guide for the delivery of the content; of note, this resource could be used as actual slides for the workshop, if desired. The facilitator first prompted participants to think about a time when they had been misunderstood or had misunderstood someone else, encouraging dramatic or humorous examples. The facilitator went on to set the context for how incorrect assumptions are made, presented the goals of the workshop, introduced the Ladder of Inference and each of its components, and summarized at the end. Personal anecdotes from the facilitator and participants were encouraged throughout this phase, which took approximately 30 minutes.

Exercise 1: Same Situation, Characters Vary proceeded as laid out in detail in Appendix G. Each group moved to a separate section of the room where a visual representation of the Ladder of Inference (Appendix A or B) had been pre-positioned, as well as one set of Rung Concept Cards (Appendix E) for every round planned for the activity. The activity proceeded as guided by the facilitator according to Appendix G. Briefly, each group received a Character Card describing plausible traits, accomplishments, or recent experiences of a fictitious resident-character (Appendix C). Instructed to imagine themselves as that character, each group was presented with the same intentionally ambiguous inciting event. Specifically, the groups were told to imagine that their character had just received an email saying they were to meet with their program director that afternoon, with no further context. Given this selected piece of reality, groups then interpreted the message, made assumptions, and so on, proceeding all the way up the Ladder of Inference framework, writing their inferences on the correspondingly titled Rung Cards at their stations, and attaching those Rung Cards to the ladder posters. Once all the groups had finished, they reported out the inferences they had made and the actions taken by their hypothetical character. Moving to small groups, instruction, and distribution of Character Cards took 10 minutes. Then, each round of exercise 1 took about 20 minutes.

With the small groups remaining at their stations, the facilitator proceeded with exercise 2: Different Situations (Appendix H). Rather than Character Cards, each group received a Situation Card describing a fictitious situation that might be faced by any person, with specific character traits being less important than in exercise 1. These Situation Cards (Appendix D) described emotionally charged scenarios that could be interpreted any number of ways. Some were relevant to the residency environment, and others were not. The activity proceeded otherwise as exercise 1 had. Moving to small groups, instruction, and distribution of Situation Cards took 5 minutes. Then, each round of exercise 2 took about 20 minutes.

Upon conclusion of exercise 2, the whole group reconvened at the circle of chairs. The second half of Appendix F (slides 17-24) presented a guide for how the debrief should be run and similarly could be used to structure the discussion, if desired. First, the facilitator encouraged the participants to discuss their observations and reflections. Then, the facilitator asked them to consider means whereby they could mitigate some of the potentially negative outcomes of making inferences. Using the slides in Appendix F, the facilitator ensured that the participants considered reflecting, telling, and asking as methods whereby they could improve communication and achieve more accurate and potentially positive communication. Following this debrief and instruction, participants could return to their small-group stations and apply their knowledge by playing the roles of the

Copyright © 2020 Schlegel and Parascando. This is an open-access publication distributed under the terms of the Creative Commons Attribution license.

two parties in conflict from exercise 2 and utilizing the reflecting, asking, and telling strategies as practice. A final summary of the Ladder of Inference concept and how to improve communication concluded the presentation. This education and reflection took 35 minutes. The entire workshop lasted 2 hours, or longer if second rounds were done for any exercises.

Assessment

This study was approved by the Penn State College of Medicine Institutional Review Board through expedited review and was considered not to be human research.

For both cohort A and cohort B, all attendees received a paper copy of the assessment immediately following the workshop. The 12-item assessment was a modified version of the evaluation questionnaire created by Allen and Baughman.[19] We modified questions to appropriately assess the educational objectives of this workshop (Appendix I). The evaluation featured 10 questions related to workshop impressions, one on workshop satisfaction, and one open-ended question asking for additional comments and suggestions. Attendees responded to each of the 10 impression questions on a 7-point Likert scale (1 = *strongly disagree,* 7 = *strongly agree*). To index overall workshop impression, we calculated a summary score of the 10 impression questions. The satisfaction question asked about overall satisfaction with the workshop and was answered on a different 7-point Likert scale (1 = *very dissatisfied,* 7 = *very satisfied*). Demographics included year in residency, age, gender, race, and ethnicity. The assessment took approximately 5 minutes. We did not compensate attendees for completion of the assessment.

Statistical Analysis

We calculated descriptive statistics for all quantitative questions using SAS 9.4.[20] Similar to Allen and Baughman,[19] to index overall workshop impression, we calculated a summary score of the impression questions ranging from 10 to 70, with 70 being the most favorable impression. We performed correlations to determine the relationship between summary and satisfaction scores. No formal analysis was conducted for the qualitative question, as there were few responses.

Results

Cohort A

A total of 15 residents (88% response rate) completed the assessment. Table 1 presents a summary of demographic information. The majority of the respondents were male (67%), were not Hispanic or Latino (87%), were Asian (33%), and were first-year residents (33%). Table 2 summarizes the descriptive analysis of the assessment questions. The highest rating was

Table 1. Participant Demographic Information

Demographic	Cohort A	Cohort B
Age: *M* (*SD*)	29.0 (2.7)	34.6 (6.8)
Gender: no. (%)		
Female	4 (27)	10 (67)
Male	10 (67)	5 (33)
Not reported	1 (7)	0 (0)
Ethnicity: no. (%)		
Hispanic or Latino	0 (0)	4 (27)
Not Hispanic or Latino	13 (87)	9 (60)
Not reported	2 (13)	2 (13)
Race: no. (%)		
White	4 (27)	6 (40)
Black or African American	0 (0)	4 (27)
Asian	5 (33)	2 (13)
More than one race	2 (13)	0 (0)
Not reported	4 (27)	3 (20)
Year in residency: no. (%)		
First year	5 (33)	3 (20)
Second year	4 (27)	4 (27)
Third year	2 (13)	4 (27)
Fourth year	3 (20)	0 (0)
Faculty	0 (0)	1 (7)
Not reported	1 (7)	3 (20)

for the question “This workshop was effective in helping me to understand the concept of the Ladder of Inference—how people make assumptions based on incomplete information” (6.5 out of 7). The average summary score for the impression questions was 65.9 out of 70 ($SD = 9.1$). On average, the respondents found the workshop to be favorable (6.3 out of 7). Similar to Allen and Baughman,[19] we found a strong positive correlation between the single satisfaction question and the summary score from the impression questions ($r = .8$).

Five attendees responded to the qualitative question asking for comments and suggestions regarding the workshop. They provided responses such as “This was a very interactive, engaging & creative workshop,” “need more like this,” and “interactive & thought provoking!”

Cohort B

A total of 15 residents and faculty (54% response rate) completed the assessment. Table 1 presents a summary of demographic information. The majority of respondents were female (67%), were not Hispanic or Latino (60%), were Caucasian (40%), and were second- (27%) or third-year residents (27%). Table 2 summarizes the descriptive analysis of the assessment questions. The highest ratings were for the questions “This workshop captured and held my interest” (6.7 out of 7), “This workshop activity would be valuable for future residency classes in our organization” (6.7 out 7), and “This workshop activity would have value in other organizations like ours in the future” (6.7 out of 7). The average summary score for the impression questions was

Copyright © 2020 Schlegel and Parascando. This is an open-access publication distributed under the terms of the Creative Commons Attribution license.

Table 2. Descriptive Summary of Impression and Satisfaction Question Responses ($n = 15$)

Question[a]	*M* (*SD*) Cohort A	*M* (*SD*) Cohort B
1. This workshop captured and held my interest.	6.1 (1.3)	6.7 (0.6)
2. This workshop was effective in helping me to understand the concept of the Ladder of Inference—how people make assumptions based on incomplete information.	6.5 (0.5)	6.5 (0.8)
3. This workshop provided me with a means to understand the dynamics within my group as they relate to making assumptions.	6.3 (0.9)	6.6 (0.6)
4. This workshop was effective in making me explicitly aware of the detrimental impact that incomplete or incorrect information can have on a group.	6.1 (0.8)	6.5 (0.6)
5. This workshop was effective in helping me identify means to mitigate any detrimental impact that assumption making can have on a group.	6.1 (1.0)	6.3 (0.9)
6. I was able to better identify and be aware of the detrimental impact that making inaccurate or incomplete assumptions can have on interpersonal and team interactions.	6.2 (1.0)	6.5 (0.8)
7. I was able to identify techniques to minimize the problems caused by inaccurate or incomplete assumptions.	5.8 (0.9)	6.1 (1.5)
8. I plan to actively apply techniques to minimize the problems caused by inaccurate or incomplete assumptions.	6.1 (0.9)	6.2 (1.1)
9. This workshop activity would be valuable for future residency classes in our organization.	6.1 (1.6)	6.7 (0.7)
10. This workshop activity would have value in other organizations like ours in the future.	6.3 (1.6)	6.7 (0.6)
11. On a scale from 1 to 7, please indicate your overall level of satisfaction with this workshop.	6.3 (0.9)	6.8 (0.6)

[a]Questions 1-10 were rated on a 7-point Likert scale where 1 = *strongly disagree* and 7 = *strongly agree.* Question 11 was rated on a 7-point Likert scale where 1 = *very dissatisfied* and 7 = *very satisfied.*

64.8 out of 70 ($SD = 6.1$). On average, the respondents found the workshop to be favorable (6.8 out of 7). Similar to Allen and Baughman,[19] we found a strong positive correlation between the single satisfaction question and the summary score from the impression questions ($r = .9$).

Four attendees responded to the qualitative question asking for comments and suggestions about the workshop. They submitted responses such as “This was the first time in a formal setting that I was able to think through the concepts of the ladder of inference” and “This was an excellent workshop. Will definitely like to work on improving my thought process.”

A summary of the descriptive analysis of questions for both cohorts appears in Table 2. Both cohorts felt the workshop captured their interest (question 1). Responses to the questions designed to assess workshop effectiveness in helping participants understand the Ladder of Inference concept (question 2) and the dynamics within a group as they relate to making assumptions (question 3) suggest the workshop was effective. Both cohorts felt it was effective in making them aware of the detrimental impact that incomplete or incorrect information can have on a group (question 4) and on interpersonal and team interactions (question 6), as well as identifying the means to mitigate any detrimental impact that assumption making can have on a group (question 5). Responses to questions 7 and 8 suggest that following the workshop, participants were able to identify and actively apply the techniques they had learned to minimize the problems caused by inaccurate or incomplete assumptions. Both cohorts were satisfied with the workshop overall (question 11) and anticipated that it would be useful in future residency classes (question 9) and in other similar organizations (question 10). While cohort B had higher scores for the majority of survey questions, cohort A had a higher summary score (difference of 1.1). The largest difference between the cohorts was on questions 1 (0.6) and 9 (0.6).

Discussion

Psychological safety is critical to the growth and education of residents and is necessary for the collective good of a program.[4] However, residency training is rife with threats to psychological safety. We created a workshop to introduce residents to the concept of the Ladder of Inference in order to enhance psychological safety and reduce feelings of shame, defensiveness, and vulnerability. We found that the activity was effective in helping residents understand how humans take mental shortcuts that result in erroneous conclusions, which can violate psychological safety.

The very positive responses to assessment questions 1 and 2 suggest that the workshop achieved objective 1, introducing the concept of the Ladder of Inference in an engaging way. The positive responses to question 3 suggest that participants were able to recognize the applicability of the Ladder of Inference to themselves (objective 2). That participants could recognize the detrimental impact of assumptions on their program (objective 3) is suggested by the positive scores on questions 4 and 6. Finally, the positive responses to questions 5 and 7 indicate that objective 4 was met and that participants felt they could use their new insights to improve interpersonal communication in their professional roles.

Ratings for the workshop were very similar. There was a trend for ratings of the workshop to be slightly lower for cohort A as

Copyright © 2020 Schlegel and Parascando. This is an open-access publication distributed under the terms of the Creative Commons Attribution license.

compared to cohort B, although a comparison of the groups was not a primary goal of the study. This difference in ratings could potentially be due to subtle differences in the execution of the workshop, differences in the identity (e.g., specialty) of the participants, or other factors.

The Ladder of Inference has been suggested in other clinical contexts as a means of improving communication. It appears that the concept can be successfully taught to residents and faculty in the context of this workshop.

We strongly suggest that all members of the educational structure of the residency program participate in the workshop. At a minimum, this would include all residents, faculty, and the program director. Administrative staff and others who regularly interact with the residency in important ways could also be included. This could encompass clinical staff such as nurses. However, enlargement of the group by inclusion of more participants might detract from the degree of intimacy and candor of the group and should be considered in planning. Inclusion of clinical staff may so enlarge the group as to make the workshop difficult to execute and could also shift the focus of the workshop from academics to clinical considerations. This may or may not be desirable depending on the needs of the group.

We offer this workshop and the Ladder of Inference as tools for enhancing communication. Future work could measure the impact of these tools in practice. Metrics of psychological safety could be deployed before and sometime after participation in the workshop to assess effectiveness of the tool. ACGME survey results could serve as secondary markers of psychological safety. However, it must be noted that many factors intrinsic and extrinsic to a residency program can influence feelings of psychological safety, and thus, isolating the impact of the workshop may be difficult.[4]

Our study was limited by its execution within only two residency programs based in academic medical centers. Its acceptability and effectiveness could thus be a reflection of factors unique to the participants of the programs or academic medical centers, limiting generalizability. However, the similarity of responses despite the differing specialties of the residency programs suggests that the workshop could have a wider applicability. Additionally, we did not collect any data regarding the prevalence and effect of rumors in residency programs. A validated metric for assessing these aspects before and after the workshop would be very helpful in demonstrating the practical impact of the workshop.

Future research could assess the effectiveness of the workshop among other specialties and within both academic and community-based sites. The participants' opinion that the workshop would have value in other organizations suggests that it may be of use in other academic settings, such as among medical students. It could also have applicability within health care systems and organizations more broadly. In particular, the Situation and Character Cards could be adapted so that the workshop serves as an educational session dealing with issues of bias and diversity. Other applications could be developed by adapting the Situation and Character Cards to reflect local needs. For example, members of a public corporation that includes both engineering and marketing divisions could host workshops bringing together members of disparate teams. Cards could be modified to reflect the perspectives of the two divisions. Through the workshop, different teams working from disparate perspectives toward the same goal of generating revenue could come to understand one another better. Negotiations between management and union leaders could similarly benefit from an introduction to the ladder as a means of enhancing trust in pursuit of success for a business and its workers. In future deployments, the workshop itself could be modified to function via teleconference using shared screens and files rather than as an in-person event.

This study demonstrates that the Ladder of Inference is a mental model that can be effectively taught and applied within residency programs. The importance of psychological safety and the apparent effectiveness of the workshop suggest that teaching the ladder can mitigate against the stresses of residency and the corrosive effect of rumors and assumptions. By extension, the workshop could improve concrete outcomes such as increased adverse event reporting, as well as trust and morale within programs. To the degree that a residency team develops a shared understanding and application of the Ladder of Inference model, it could foster resiliency, enhance collegiality, and improve educational and clinical outcomes.

Appendices

A. Ladder of Inference Poster.pptx
B. Ladder of Inference Poster.docx
C. Character Cards.docx
D. Situation Cards.docx
E. Rung Concept Cards.docx
F. Ladder of Inference Presentation.pptx
G. Exercise 1 Instructions and Talking Points.docx

Copyright © 2020 Schlegel and Parascando. This is an open-access publication distributed under the terms of the Creative Commons Attribution license.

H. Exercise 2 Instructions and Talking Points.docx

I. Ladder of Inference Workshop Assessment Tool.docx

All appendices are peer reviewed as integral parts of the Original Publication.

Daniel Schlegel, MD, MHA: Assistant Professor, Department of Family and Community Medicine, Penn State College of Medicine

Jessica Parascando, MPH: Research Project Manager, Department of Family and Community Medicine, Penn State College of Medicine

Acknowledgments
The authors are grateful to Julie Radico, PhD, for her review of the manuscript.

Disclosures
None to report.

Funding/Support
None to report.

Prior Presentations
Schlegel D, Carey S, Givans K, Demetriou T. The story in your head: we're all climbing up the Ladder of Inference. Workshop presented at: FMEC Annual Meeting; November 1-3, 2019; Lancaster, PA.

Ethical Approval
The Penn State College of Medicine Institutional Review Board Reported approved this study.

References

1. Edmondson AC, Lei Z. Psychological safety: the history, renaissance, and future of an interpersonal construct. *Annu Rev Organ Psychol Organ Behav*. 2014;1:23-43. https://doi.org/10.1146/annurev-orgpsych-031413-091305
2. Kahn WA. Psychological conditions of personal engagement and disengagement at work. *Acad Manage J*. 1990;33(4):692-724. https://doi.org/10.5465/256287
3. Schein EH. *How Can Organizations Learn Faster? The Problem of Entering the Green Room*. Alfred P. Sloan School of Management, Massachusetts Institute of Technology; 1992. Working paper no. 3409-92.
4. Bynum WE, Haque TM. Risky business: psychological safety and the risks of learning medicine. *J Grad Med Educ*. 2016;8(5):780-782. https://doi.org/10.4300/jgme-d-16-00549.1
5. Appelbaum NP, Dow A, Mazmanian PE, Jundt DK, Appelbaum EN. The effects of power, leadership and psychological safety on resident event reporting. *Med Educ*. 2016;50(3):343-350. https://doi.org/10.1111/medu.12947
6. Holt KD, Miller RS, Philibert I, Heard JK, Nasca TJ. Residents' perspectives on the learning environment: data from the Accreditation Council for Graduate Medical Education resident survey. *Acad Med*. 2010;85(3):512-518. https://doi.org/10.1097/acm.0b013e3181ccc1db
7. Nembhard IM, Tucker AL. Deliberate learning to improve performance in dynamic service settings: evidence from hospital intensive care units. *Organ Sci*. 2011;22(4):907-922. https://doi.org/10.1287/orsc.1100.0570
8. Tucker AL. An empirical study of system improvement by frontline employees in hospital units. *Manuf Serv Oper Manag*. 2007;9(4):492-505. https://doi.org/10.1287/msom.1060.0156
9. Nefer B. Neutralizing the power of workplace gossip. *SuperVision*. 2009;70(4):14-16.
10. DiFonzo N, Bordia P, Rosnow RL. Reining in rumors. *Organ Dyn*. 1994;23(1):47-62. https://doi.org/10.1016/0090-2616(94)90087-6
11. Rahmani M. Helping program directors effectively manage rumors and gossip. *J Grad Med Educ*. 2018;10(6):616-619. https://doi.org/10.4300/JGME-D-18-00380.1
12. Ribeiro VE, Blakeley JA. The proactive management of rumor and gossip. *J Nurs Adm*. 1995;25(6):43-50. https://doi.org/10.1097/00005110-199506000-00010
13. Chase P, Stuart GW. Rumor management in nursing systems: role of the psychiatric CNS. *Clin Nurse Spec*. 1995;9(6):327-332. https://doi.org/10.1097/00002800-199511000-00015
14. Gregg RE. Office gossip: a surprising source of liability. *J Med Prac Manage*. 2003;19(2):71-74.
15. Argyris C. *Overcoming Organizational Defenses: Facilitating Organizational Learning*. Allyn and Bacon; 1990.
16. Pilette PC. Collaborative capital: conversation for a change. *Nurs Manage*. 2006;37(11):24-28. https://doi.org/10.1097/00006247-200611000-00007
17. Senge PM, Kleiner A, Roberts C, Ross RB, Smith BJ. *The Fifth Discipline Fieldbook: Strategies and Tools for Building a Learning Organization*. Currency, Doubleday; 2006:242-246.
18. Ridings A. *Pause for Breath: Bringing the Practices of Mindfulness and Dialogue to Leadership Conversations*. Troubador Publishing; 2020:75-100.
19. Allen PJ, Baughman FD. Active learning in research methods classes is associated with higher knowledge and confidence, though not evaluations or satisfaction. *Front Psychol*. 2016;7:279. https://doi.org/10.3389/fpsyg.2016.00279
20. SAS Institute. *Base SAS 9.4 Procedures Guide*. SAS Institute; 2015.

Received: February 11, 2020
Accepted: July 30, 2020
Published: November 30, 2020

Copyright © 2020 Schlegel and Parascando. This is an open-access publication distributed under the terms of the Creative Commons Attribution license.
